# Cdc42 and Rac2 act through the formin-like Frl/FMNL to control lamellocyte shape and encapsulation of parasitoid wasp eggs in *Drosophila*

**DOI:** 10.1371/journal.ppat.1014440

**Published:** 2026-07-20

**Authors:** Darius Molitor, Hermann Schillers, Sven Bogdan

**Affiliations:** 1 Institute of Physiology and Pathophysiology, Department of Molecular Cell Physiology, Philipps University Marburg, Marburg, Germany; 2 Institute of Physiology II, University of Münster, Münster, Germany; University of Kansas, UNITED STATES OF AMERICA

## Abstract

*Drosophila* has been established as a powerful genetic model to study not only blood cell development but also innate cellular immunity. Lamellocytes are the most distinct blood cell type which are large key effector cells in the anti-parasitoid immune response in *Drosophila.* Findings on cell morphology and function of lamellocytes are based on pioneering studies conducted in the early 1980s. Here, we analyzed their remarkable cell cytoskeleton and cell morphology using high-resolution microscopy combined with a lamellocyte-specific candidate RNAi approach to identify key regulators of the lamellocyte morphology and functions. Similar to disc-shaped rigid platelets in our bloodstream, non-migratory lamellocytes only passively circulate in the hemolymph. Once attached to parasitic wasp egg, lamellocytes undergo a marked reorganization of their cortical actin cytoskeleton promoting cell spreading and encapsulation of parasitic wasp eggs. Atomic force microscopy (AFM*)* indeed showed that lamellocytes become significantly softer while the cortical actin cytoskeleton reorganizes and forms lamellipodia-like protrusions to spread. Cortical actin cytoskeleton reorganization does not depend on WAVE-Arp2/3-branched actin filament nucleation but rather requires the formin Frl (also known as FMNL) downstream of Rac2 and Cdc42 signaling. Supporting this notion, RNAi-mediated depletion of either Frl/FMNL or Rac2 and Cdc42 but not Rac1 results in prominent changes in lamellocyte morphology and immune dysfunction. Our data further suggest a pathway in which Rac2/Cdc42 recruits Frl/FMNL to the cell cortex controlling lamellocyte spreading and encapsulation of parasitoid wasps.

## Author summary

Lamellocytes are giant immune cells that play a crucial role in defending against parasitoid wasp infection in the fruit fly *Drosophila*. While they are barely present in healthy animals, they are rapidly induced in response to infection and enclose the wasp egg in a cellular capsule. Despite this fascinating mechanism, many aspects of the lamellocyte cell biology, including the regulation of their unique cortical actin-cytoskeleton remain poorly understood. Using high-resolution imaging, we show that non-motile lamellocytes are very rigid compared to migratory macrophage-like plasmatocytes but get softer when attached to surfaces by reorganization of their cortical actin cytoskeleton. In a RNAi candidate approach, we found that attachment and spreading of lamellocytes does not depend on the WAVE regulatory complex and its main effector Arp2/3 complex, but on the formin FMNL/Frl. This conserved actin nucleator is likely regulated and recruited by the small GTPases Rac2 and Cdc42. In conclusion, we provide a detailed characterization of the lamellocyte cytoskeleton and identified a pathway in which Rac2/Cdc42 recruits Frl/FMNL to the cell cortex controlling lamellocyte spreading and encapsulation of parasitoid wasps.

## Introduction

Immune cells highly depend on rapid cytoskeletal remodeling, both in innate and the adaptive immune responses to infections. The actin cytoskeleton is especially important for normal immune cell shape as well as for essential functions in immune defense, including migration, phagocytosis, activation, secretion, and cell-cell adhesion. Many of these cytoskeletal processes are common to diverse cell types, their regulators, particularly those driving the cortical actin cytoskeleton, are often specific for distinct immune effector cells or hematopoietic lineages. Mutations in cytoskeletal regulatory proteins often compromise distinct cellular aspects of the immune response and immunopathies which serve as genetic models to study the regulation of the actin cytoskeleton [1]. *Drosophila* has been established as powerful genetic model to study not only blood cell development but also innate cellular immunity [2–5]. Three main blood cell types have been described in *Drosophila* which are analogous to vertebrate myeloid cells. Plasmatocytes, the main immune cell type in healthy larvae, are professional macrophages, whereas crystal cells only account for ~5% of the larval blood population involved in the melanization/clotting reaction. The third cell type, lamellocytes are the most morphologically distinct blood cell type which only differentiate upon wasp parasitization in *Drosophila* [5–8]. These leaflike, extremely large (up to 100 µm in diameter) cells are ideally suited for their function, which is to wrap large parasitoid eggs deposited into the larval body cavity [9,10]. Together with crystal cells and plasmatocytes, they form a multilayered and rigid melanized capsule which leads to the death of the invading parasitoid [11]. Lamellocyte encapsulation is a multiple step process that requires cell attachment, spreading on the wasp egg and the formation of cellular junctions effectively separating the wasp egg from the host [12,13]. These fundamental cellular processes involve dynamic changes in lamellocyte shape that require a tight regulation of their cytoskeleton. Recent proteomic and transcriptomic studies revealed several integrins, integrin-associated proteins, actin-binding and microtubule-binding proteins that are enriched in lamellocytes and might be involved in lamellocyte shape and cytoskeletal dynamics [14,15]. While previous research has focused on the differentiation of lamellocytes, the relationship between the cytoskeleton of lamellocytes and their functions in host defense has not yet been studied in detail [2].

Here, we analyzed the unique actin cytoskeleton of these specialized immune effector cells. Circulating, giant lamellocytes have a dense actin filament network with no distinct lamellipodial regions at the cell cortex. Once attached, lamellocytes undergo a marked reorganization of their cortical actin cytoskeleton promoting cell spreading and encapsulation of parasitic wasp egg. Isolated lamellocytes are capable of forming dynamic membrane protrusions when spread on ConA-coated surfaces. Spread lamellocytes form thin, non-canonical sheet-like lamellipodial protrusions which do not require WRC-Arp2/3-branched actin nucleation. Instead, we identified the Rho GTPases Rac2 and Cdc42 as well as the formin Frl, the single fly member of the FMNL (formin related in leukocytes/formin-like) formin subfamily as key regulators controlling lamellocyte actin cytoskeleton and cell shape. Both, Rac2 and Cdc42 control the localization of Frl/FMNL at lamellocyte cortex. Infection experiments further revealed that Rac2 and Frl/FMNL are necessary for the proper encapsulation of eggs from the parasitoid wasp *L. boulardi*. Our data propose a model in which Cdc42 together with Rac2 acts through Frl/FMNL to control lamellocyte shape and, as a consequence, the effectivity of the encapsulation response against parasitoid wasp eggs*.*

## Results

### Changes in the stiffness and morphology of lamellocytes during cell spreading are closely linked to the reorganization of the cortical actin cytoskeleton

**Fig 1:**
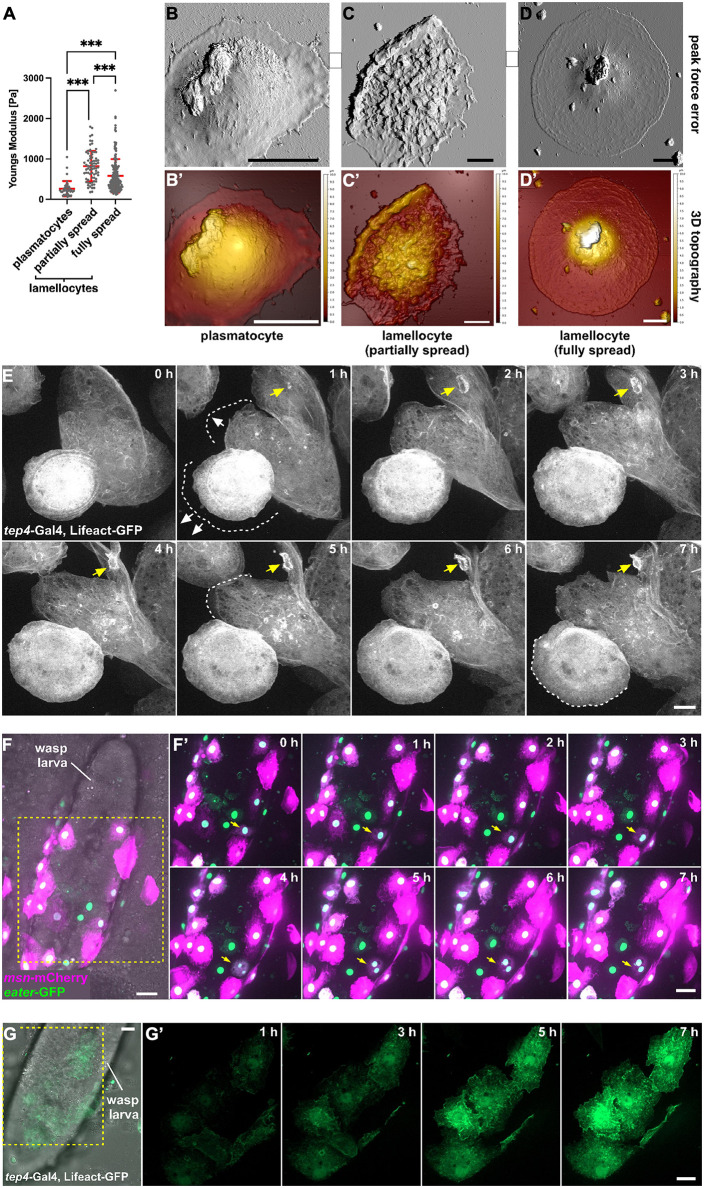
Lamellocyte stiffness is linked to rearrangements of the cortical actin cytoskeleton. **(A)** Elasticity of cells given as the Young`s modulus. Indentation experiments revealed that partially spread lamellocytes are stiffer than fully spread lamellocytes. N = 40 (plasmatocytes), 74 (partially spread lamellocytes) and 180 (fully spread lamellocytes) force-distance curves (technical replicates from all experiments) from 3 independent biological replicates (separate vials, 10-15 larvae each); One-way ANOVA with Kruskal-Wallis test, ***: p < 0.001. Mean values: 261.58 Pa ± 189.13 (plasmatocytes); 820.95 Pa ± 374.14 (partially attached lamellocytes) and 581.85 Pa ± 414.08 (fully attached lamellocytes). **(B-D’)** Images of individual cells obtained in PeakForce Tapping mode. The dimensions of the images are as follows: 10 µm in height and lateral expansion of 25 x 25 µm for B and B’, and 65 x 65 µm for C, C’, D and D’. **(B-D)** show grey-scale coded peak force error images, a high contrast channel with precise lateral dimensions but lacking height information. **(B’-D’)** show a 3D view of individual cells. The heights are color-coded and relate to the vertical scale bar. The Concanavalin A-coated surface appears as a red plane beneath the cell bodies. Cells show different morphologies and sizes. Plasmatocytes (B, B’) are rather small with a maximum height of 5.5 µm compared to the partially spread (C, C’; 8.2 µm) and fully spread (D, D’; 9.9µm) lamellocytes. Scale bars: 10 µm. **(E)** Still frames from a timelapse movie of lamellocytes spreading on Concanavalin A-coated glass. The actin cytoskeleton is labelled by Lifeact-GFP under the control of *Tep4*-Gal4. Scale bar: 10 µm. Representative results from more than 10 experiments. **(F)** Still frame from a timelapse movie showing a parasitoid wasp egg with attached hemocytes. Plasmatocytes are marked by *eater*-GFP-NLS, while lamellocytes express *msn*-mCherry. Frequently, lamellocytes are positive for both markers. Scale bar: 10 µm. Representative results from more than 10 separate crossings/vials as biological replicates. **(F’)** Magnifications of the dashed area in **F.** An intermedate cell expressing mCherry and GFP-NLS can be observed dividing on the wasp egg (yellow arrow). Scale bar: 10 µm. **(G)** Still frame from a timelapse movie. Lamellocytes are attached to a parasitoid wasp egg, the actin cytoskeleton is marked by Lifeact-GFP under the control of *Tep4*-Gal4. Scale bar: 10 µm. Representative results from more than 10 separate crossings/vials as biological replicates. **(G’)** Magnifications of the dashed area in G. Expression levels of *tep4*-Gal4 increase over the course of several hours of spreading. Scale bar: 10 µm.

*Drosophila* lamellocytes have been described as large, flat, F-actin-rich immune cells that form in response to stress, such as parasitic wasp infections [10,16]. To visualize lamellocytes we bled wandering third-instar larvae expressing a Lifeact-GFP transgene under the control of the Tep4-Gal4 driver which had been previously been infected by parasitic wasps. Different from the smaller, highly motile macrophage-like plasmatocytes, isolated lamellocytes have a rigid disc-shaped morphology with no prominent protrusion dynamics when plated on glass surfaces (S1 Movie). To better characterize the mechanical properties of single lamellocytes, we performed nanoindentation experiments using atomic force microscopy (AFM) which is the most effective method for determining the mechanical properties of soft biological materials and biomaterials at the nanoscale [17]. In detail, we measured the elasticity of spread lamellocytes given as the Young’s modulus (Fig 1A). Young’s modulus quantifies a cell’s stiffness by measuring how much it deforms under an applied stress, so a lower Young’s modulus indicates a more elastic (softer) cell and a higher Young’s modulus indicates a less elastic (stiffer) cell. These indentation experiments revealed that lamellocytes are more rigid than smaller plasmatocytes (261.58 Pa ± 189.134; Fig 1A, 1B). Moreover, these measurements also revealed that partially spread lamellocytes (820.95 Pa ± 374.14) are significantly stiffer than lamellocytes fully spread on Concanavalin A (581.85 Pa ± 414.08; Fig 1A, 1C, 1D). Changes in lamellocyte stiffness are closely linked to dramatic changes in their cell morphology. Lamellocytes, which were only partially spread, exhibited numerous membrane ruffles on their dorsal surface (Fig 1C, 1C’). By contrast, lamellocytes fully spread on Concanavalin A, were extremely thin without prominent membrane ruffles (Fig 1D, 1D’). A rigid disc shaped-morphology of lamellocytes can also be seen in living third instar larvae, where they circulate passively in the hemocoel with the hemolymph (S2 Movie). Changes in lamellocyte rigidity and morphology are likely driven by changes of the cortical actin cytoskeleton. A prominent reorganization of the cortical actin cytoskeleton was observed in isolated lamellocytes expressing Lifeact-GFP as a reporter for visualizing F-actin structures over several hours (S3 Movie). Spreading lamellocytes formed lamellipodia-like protrusions at the cell periphery, but also exhibited large circular membrane ruffles and vesicles with intense ring-like F-actin accumulations (Fig 1E; S3 and S4 Movies). We next imaged lamellocyte directly spreading on parasitoid wasp eggs isolated from infested L2 fly larvae expressing a dual reporter msn-Cherry/eaterGFP reporter *ex vivo* in dissection medium. This dual reporter marks not only lamellocyte lineages (cytoplasmic mCherry expression) but also the plasmatocyte lineage (nuclear GFP) that transdifferentiates into lamellocyte-like cells [12]. We found both lineages, lamellocytes (mCherry positive), plasmatocytes (eater-GFP) as well as intermediates expressing both markers attached to the eggs (Fig 1F, 1F’; S5 Movie). Once attached to a developing parasitic wasp larva, mCherry-positive spread on the wasp eggs and underwent a similar change in cell morphology like isolated lamellocytes plated on Concanavalin A (Fig 1F, 1F’; S5 Movie). Some intermediates were dividing while attached to a wasp egg as previously reported [12]. Spreading lamellocytes expressing the LifeAct-GFP reporter formed dynamic thin F-actin-rich membrane protrusions and vesicles (Fig 1G, 1G’; S6 Movie).

**Fig 2:**
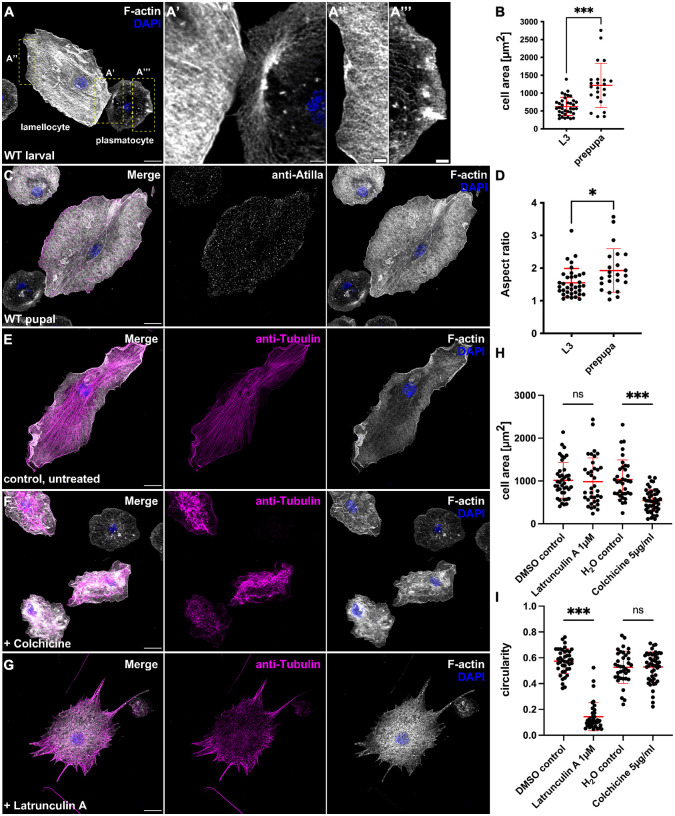
Morphology and actin cytoskeleton of *Drosophila* lamellocytes. **(A)** Confocal microscopy image of a lamellocyte (left) and a macrophage-like plasmatocyte (right) isolated from a wasp-infested wild type L3 instar larva. F-actin is stained by phalloidin (white) and nuclei by DAPI (blue), scale bar: 10 µm. **(A’-A”’)** Magnifications of areas indicated in A. Note the lamellipodium, consisting of branched actin filaments, in plasmatocytes compared to the dense cortical meshwork in lamellocytes, which lacks morphologically distinct substructures. Scale bars: 2 µm. Representative images from 3 independent experiments/biological replicates with 10 larvae each. Representative results from more than 10 separate crossings/vials as biological replicates. **(B)** Quantification of cell size between larval (L3) and prepupal lamellocytes. N = 36 (L3) and 23 cells (prepupa; technical replicates from all experiments) from 3 independent biological replicates from separate vials, 10 animals each); Mann-Whitney test, ***: p < 0.001. **(C)** Confocal microscopy image of hemocytes from wasp-infested prepupae, stained with an antibody against the lamellocyte-specific marker Atilla (magenta). F-actin is visualized by phalloidin (white) and nuclei with DAPI (blue). Scale bar: 10 µm. Representative image from 3 independent biological replicates from separate vials, 10 animals each. **(D)** Quantification of aspect ratios (ratio between longest and shortest axis of the cell) between lamellocytes isolated from larvae (L3) and prepupae, showing that pupal lamellocytes are significantly more elongated that those from larvae. N = 36 (L3) and N = 23 cells (prepupa; technical replicates from all experiments) from three independent experiments (biological replicates from separate vials, 10 animals each, same cells as in B); Mann-Whitney test, *: p = 0.0125. **(E)** Confocal microscopy image of a lamellocyte. Microtubules are visualized with an anti-β-Tubulin antibody (magenta). F-actin is stained by phalloidin (grey) and the nucleus with DAPI (blue). Scale bar: 10 µm. Representative image from 3 independent biological replicates from separate vials, 10 animals each **(F)** Lamellocytes treated with the microtubule-depolymerizing drug colchicine (5 µg/ml, for 1 hour). Note the reduced cell size, crinkled cell surface and disordered anti-β-Tubulin staining (magenta) compared to the untreated control in E. F-actin is stained by phalloidin (grey) and the nucleus with DAPI (blue). Scale bar: 10 µm. Representative image from 3 independent biological replicates from separate vials, 10 animals each. **(G)** A lamellocyte treated with the F-actin-depolymerizing drug latrunculin A (1µM, for 1 hour). The cell displays a stellate shape with long, microtubule-rich protrusions. Anti-β-Tubulin is visualized by antibody staining (magenta), F-actin with phalloidin (grey) and the nucleus with DAPI (blue). Scale bar: 10 µm. Representative image from 3 independent biological replicates from separate vials, 10 animals each. **(H)** Comparison of cell area between drug treatments and controls. Treatment with colchicine causes a significant reduction of cell size compared to the water control while the cell size is unchanged by latrunculin A treatment. N = 41 (DMSO control), N = 36 (latrunculin and H_2_O control) and N = 48 cells (colchicine; technical replicates from all experiments) from 3 independent biological replicates from separate vials, 10 animals each. One-way ANOVA with Kruskal-Wallis test, ns: p > 0.999; ***: p < 0.001. **I)** Quantification of the cell circularity between drug treatments and controls. Latrunculin A-treatment causes a drastic reduction in cell circularity compared to the control. Treatment with colchicine on the other hand does not significantly change the cell circularity. N = 41 (DMSO control), N = 36 (latrunculin and H_2_O control) and N = 48 cells (colchicine; technical replicates from all experiments) from three independent 3 independent biological replicates from separate vials, 10 animals each; same cells as in **H)**; One-way ANOVA with Kruskal-Wallis test, ***: p < 0.001; ns: p > 0.999.

We next analyzed the lamellocyte spread morphology in more detail by high-resolution confocal microscopy (Fig 2). For quantitative analysis, we also took advantage of *hop*^*Tum-l*^ mutant larvae bearing a dominant gain-of-function mutation in the JAK/STAT pathway that constitutively produces large numbers of lamellocytes [18]. Isolated lamellocytes spread on Concanavalin A were stained with phalloidin to visualize their actin cytoskeleton and cell shape at high resolution (Fig 2). Large lamellocytes ranging in diameter up to 100 µm can be easily identified using the molecular marker Atilla-L1, a GPI-anchored protein found on the surface of lamellocytes [19]. Compared to the much smaller macrophage-like plasmatocytes, lamellocytes have a much denser actin filament network with no distinct lamellipodial regions at the cell cortex (Fig 2A). Atilla-positive lamellocytes from prepupa were more elongated and significantly larger compared to lamellocytes isolated from third instar larvae (Fig 2B, 2C, 2D). Spread lamellocytes also showed prominent microtubule arrays from the perinuclear region radiating toward the cell periphery (Fig 2E). Disruption of the microtubule network by colchicine caused a significant cell shrinkage (Fig 2F; quantification in Fig 2H). In addition to reduced cell size, we observed a crinkling of lamellocyte surfaces (Fig 2F; quantification in Fig 2I; S7 Movie). By contrast, treatment with Latrunculin A (LatA), a widely used toxin used to depolymerize actin filaments resulted in marked changes in lamellocyte shape (Fig 2G; S7 Movie). LatA-treated lamellocytes developed a stellate-shaped morphology with numerous filamentous extensions enriched with arrays of microtubules (Fig 2G, quantification in Fig 2I; S7 Movie). Taken together, the actin and microtubule cytoskeleton contribute significantly to the morphology and differentially impact on lamellocyte shape and size.

### Genetic and pharmacological inhibition of either Arp2/3 complex or WAVE in lamellocytes did not affect the actin cytoskeletal organization and cell shape

**Fig 3:**
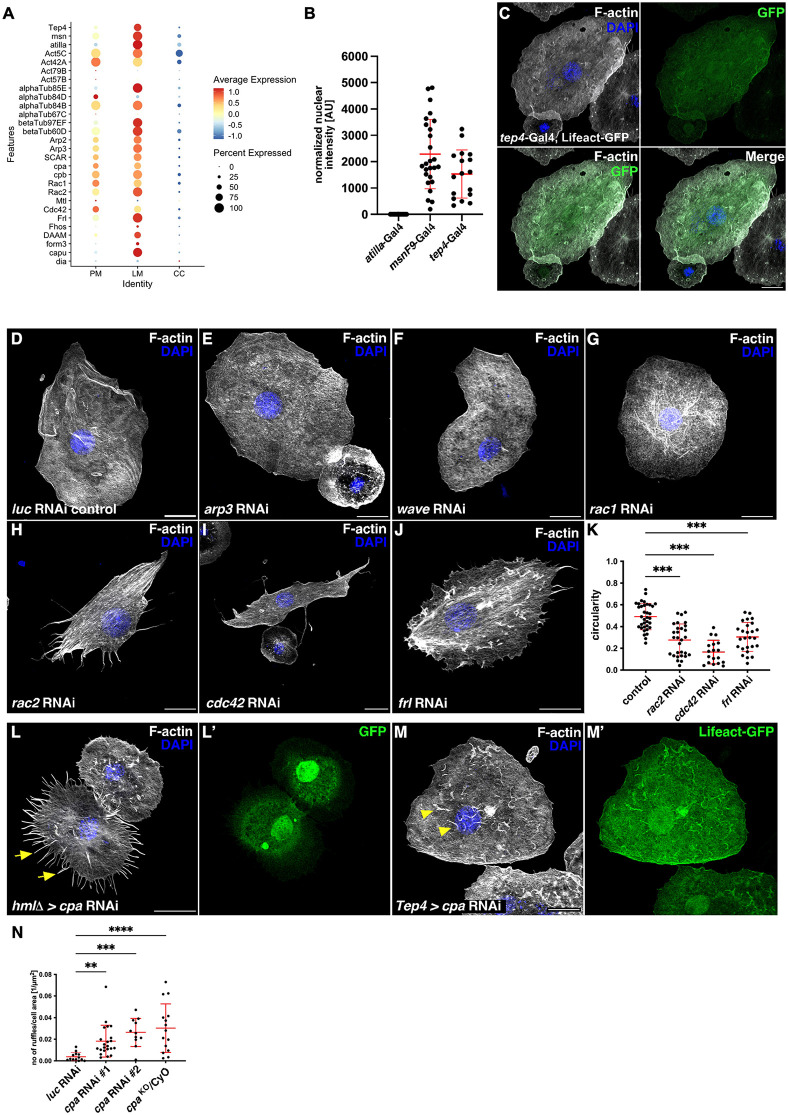
Cell type-specific RNAi-mediated knockdown of candidate genes reveals a role of Frl/FMNL in lamellocyte cell shape control. **(A)** Dotplot comparing expression levels of common lamellocyte marker genes, different isoforms of actin and tubulin and selected cytoskeletal regulators between plasmatocytes (PM), lamellocytes (LM) and crystal cells (CC), taken from a previously published scRNA data set [20]. Color shades indicate average expression levels of genes in the respective cell type. Dot sizes (“% expressed”) indicate the relative number of cells where the genes were detected. **(B)** Quantification of relative Gal4 driver strengths, measured by the intensity of a fluorescent protein fused to a nuclear localization signal (NLS-mCherry). N = 38 (*atilla*-Gal4), N = 26 (*msnF9*-Gal4) and N = 17 cells (*tep4*-Gal4; technical replicates from all experiments) from three independent biological replicates from separate vials, 10 animals each. (**C)** Confocal microscopy image of a lamellocyte expressing the F-actin probe Lifeact-GFP under control of the *tep4*-Gal4 driver, allowing for cell type-specific targeting of RNAi transgenes. Scale bar: 10 µm. Representative image from 3 independent biological replicates from separate vials, 10 animals each. **(D-J)** Lamellocyte cell morphology under different RNAi-mediated knockdown situations using *tep4*-Gal4. Representative image from 3 independent biological replicates from separate vials, 10 animals each. **(D)** RNAi against *luciferase* as a control condition. **(E)** RNAi-mediated knockdown of *arp3*, **(F)** *wave* and **(G)** *rac1*. No obvious changes in cell shape or actin organization can be observed in either case. **(H)** Knockdown of *rac2*, **(I)** *cdc42* and **(J)** *frl* in lamellocytes on the other hand results in a stellate cell shape. F-actin is stained with phalloidin (grey) and nuclei with DAPI (blue). Scale bars: 10 µm. **(K)** Quantification of the reduction of lamellocyte cell circularity caused by RNAi-mediated knockdown. N = 35 (control), N = 29 (*rac2* RNAi), N = 18 (*cdc42* RNAi) and N = 26 cells (*frl* RNAi; technical replicates from all experiments) from at least 3 independent biological replicates from separate vials, 10 animals each; ***: p < 0.001. **(L)** Stellate protrusions (arrows) in a plasmatocyte, caused by RNAi-mediated knockdown of *cpa* under the control of *hemolectin(hmlΔ)*-Gal4. F-actin is stained by phalloidin (grey) and the nuclei with DAPI (blue). Scale bar: 10 µm. Representative image from 3 independent biological replicates from separate vials, 10 animals each. **(L’)** GFP marking expression of the *hmlΔ*-Gal4 driver. **(M)** The same RNAi causes extensive dorsal ruffles (arrowheads) in lamellocytes when under the control of *tep4*-Gal4. F-actin is stained by phalloidin (grey) and the nuclei with DAPI (blue). Scale bar: 10 µm. Representative image from 3 independent biological replicates from separate vials, 10 animals each. (**M’)** Lifeact-GFP marks expression of the *tep4*-Gal4 driver. **(N)** Quantification showing significantly more dorsal ruffles per cell in *cpa*-knockdown and -knockout lamellocytes than controls. N = 13 (control), 22 (RNAi #1), N = 11 (RNAi #2) and N = 15 cells (*cpa*^*KO*^*/CyO*; technical replicates from all experiments) from 3 independent biological replicates from separate vials, 10 animals each; **: p = 0.001; ***: p < 0.001.

Proteomics identified new cytoskeletal regulators with possible cellular functions in lamellocytes [15]. Single-cell transcriptomics further confirmed a few differentially expressed tubulin and actin isoforms but also prominent actin regulators such as members of the Arp2/3 complex, SCAR/WAVE regulatory complex members, Rho GTPases and diverse formins enriched in lamellocytes [20] Fig 3A). To dissect the role of these actin regulators in the cytoskeletal organization and morphology of lamellocytes we performed a lamellocyte-specific RNAi candidate screen using the *Tep4*-Gal4 driver. Different from the two most commonly used lamellocyte driver lines, Atilla (L1) and Misshapen (Msn), *Tep4*-Gal4 is active early on in prohemocytes and later in the lamellocyte lineage, where it is expressed at significantly higher levels than *Atilla*-Gal4 (Fig 3B) [21,22,23]. We tested two or more independent RNAi lines per candidate gene under the control of the *Tep4*-Gal4 driver (see also list in S1A Fig). We first analyzed the contribution of the WAVE-Arp2/3 pathway in regulating lamellocyte morphology. Numerous studies identified WAVE-Arp2/3 branched actin nucleation as a universal key driver in lamellipodial protrusions and cell shape in many eukaryotic cells including *Drosophila* macrophage-like plasmatocytes [24–26]. A plasmatocyte-specific RNAi-mediated knockdown of wave or Arp2/3 complex subunits using the *hmlΔ*-Gal4 driver resulted in prominent defective cell morphology as previously reported (S1B Fig, B’) [26]. Members of the WRC and Arp2/3 are also highly expressed in lamellocytes (Figs 3A, S1C, S1C’). However, depletion of neither WAVE nor Arp2/3 complex components in lamellocytes affected the actin cytoskeletal organization and cell shape compared to controls (Figs 3D, 3E, 3F; S1D, D’). Similarly, pharmacological inhibition of the Arp2/3 complex using the small-molecule inhibitor CK666 [27] only resulted in plasmatocytes in a star-like cell phenotype, but not in lamellocytes (S1E, S1F Fig).

### Frl/FMNL, Cdc42 and Rac2 are required for lamellocyte shape

We next analyzed the role of Rho-family GTPases as central molecular switches to control actin organization and dynamics in eukaryotic cells [28]. RNAi-mediated knock-down of Rho GTPases Cdc42, Rac2 but not Rac1 dramatically changed lamellocyte morphology (Fig 3G-3I). Compared to controls, cells take on a stellate-shaped form, comparable to LatA-treated cells (Fig 3G-3I). Interestingly, a similar phenotype was observed upon depletion of the formin Frl/FMNL, the single fly member of the FMNL (formin related in leukocytes/formin-like) formin subfamily [29–31]. The phenotypic analysis revealed a similarly reduced circularity in lamellocytes depleted for Rac2, Cdc42 and Frl/FMNL compared to controls (Fig 3K). None of these RNAi-mediated knockdowns significantly alter hemocyte identities or the expression level of the *Tep4*-Gal4 driver in lamellocytes (S1G Fig).

More subtle changes in lamellocyte morphology were found upon depletion of Spire, and the formins Capuccino and Fhos/Knittrig (S1H, S1I, S1J Fig). No changes in lamellocyte shape were found upon depletion of two additional conserved formins, namely, Dia and DAAM (Dishevelled-associated activator of morphogenesis) which are expressed as strongly as Frl/FMNL in lamellocytes (Fig 3A). This suggests that Frl/FMNL might play a particularly important role in actin-driven lamellocyte morphology. An additional candidate gene we found in our screen that significantly controls the cell morphology of lamellocytes is the capping protein alpha (Cpa) which encodes an actin-binding protein that binds to the fast-growing ends of actin filaments to restrict the addition or loss of actin monomers [32]. Surprisingly, depletion of Cpa in lamellocytes did not lead to increased filopodia formation, as is observed in many cells as well as in plasmatocytes (Fig 3L, 3L’; [26,33]). Instead, lamellocytes depleted for Cpa showed an intense membrane ruffling at the dorsal surface of lamellocytes (Fig 3M, 3M’ and S8 Movie). These circular dorsal membrane ruffles were also observed in lamellocytes isolated from heterozygous *cpa* mutant larvae (see quantification in Fig 3N). Thus, actin dynamics regulate not only cell spreading but also membrane ruffling and both features might contribute to lamellocyte function during the encapsulation response.

### The localization of Frl/FMNL at the cell cortex depends on Cdc42 and Rac2 functions

**Fig 4:**
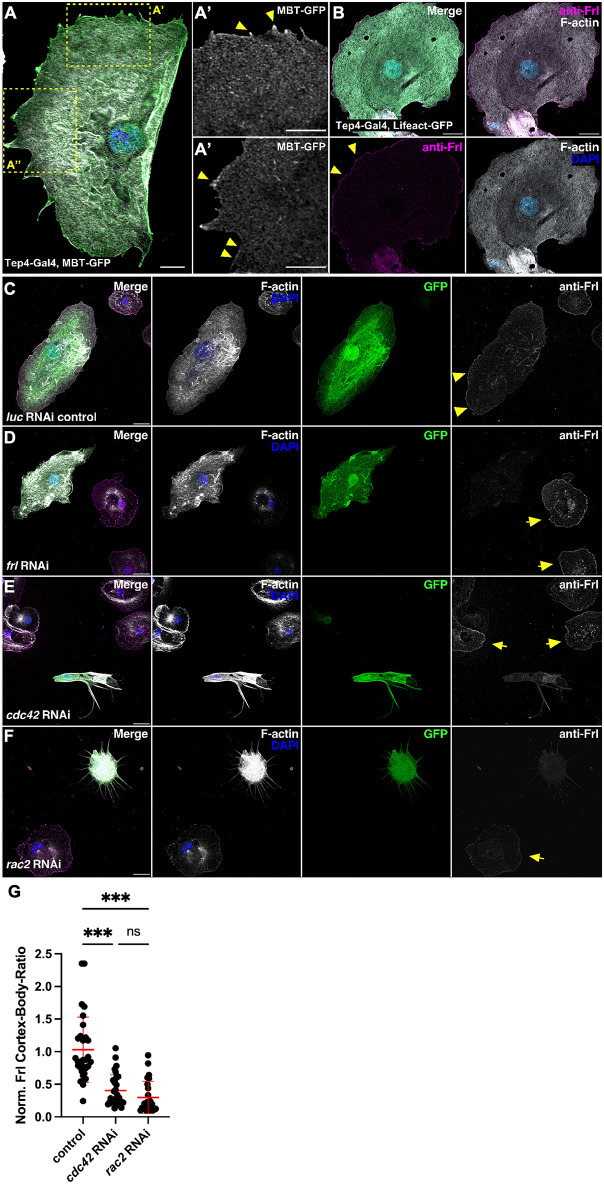
Localization of Frl/FMNL at the lamellocyte cortex depends on both Rac2 and Cdc42. **(A)** Confocal microscopy image of a lamellocyte expressing the Rac/Cdc42-sensor MBT-GFP. F-actin is visualized with phalloidin (grey) and nuclei with DAPI (blue). Scale bar: 10 µm Representative image from 3 independent biological replicates from separate vials, 10 animals each. **(A’, A”)** Magnified views from A. MBT-GFP localizes to the cell cortex and protrusion tips. Scale bars: 10 µm. **(B)** High resolution confocal microscopy image of a wildtype lamellocyte showing a cortical expression of Frl/FMNL. Frl is stained with a specific antibody (magenta), F-actin with phalloidin (grey) and the nucleus with DAPI (blue). Lifeact-GFP marks expression of the *Tep4*-Gal4 driver. Representative image from 3 independent biological replicates from separate vials, 10 animals each. **(C)** Lamellocyte expressing *luciferase* RNAi driven by *Tep4*-Gal4 as a control. Again, the cortical expression of Frl is evident (arrowheads). Representative image from 3 independent biological replicates, 10 animals each. **(D)** This localization is almost completely lost in *frl* RNAi lamellocytes, but not in plasmatocytes, where the driver is not active (arrows), confirming specificity of the RNAi and the antibody. Representative image from 3 independent biological replicates from separate vials, 10 animals each. **(E)** The cortical localization of Frl/FMNL is also drastically reduced in *cdc42* knockdown and **(F)** *rac2* knockdown lamellocytes, but not in plasmatocytes, where the driver is again not active (arrows). Scale bars: 10 µm. Representative images from 3 independent biological replicates from separate vials, 10 animals each. **(G)** Quantification of the loss of cortical Frl/FMNL localization. Both *cdc42* and *rac2* knockdown cells display a reduced cortex:body-ratio of Frl/FMNL intensity (reduced cortical localization of Frl). N = 30 (control), N = 31 (*cdc42* RNAi) and N = 25 cells (*rac2* RNAi; technical replicates from all experiments) from 3 independent biological replicates, 10 animals each; ***: p < 0.001.

Frl/FMNL formins have been originally described as *formin-related gene in leukocytes* (FRL/FMNL1) that bind Rac1 and regulate cell motility and survival of macrophages [34]. Later studies have further shown that FMNL subfamily formins FMNL2 and FMNL3 are rather Cdc42 effectors to promote protrusion efficiency and actin assembly in cells, even in the absence of the WAVE regulatory complex (WRC) [35–37]. Different from vertebrates, the fly genome only contains one representative of the FMNL subfamily, Frl/FMNL that strongly binds to GTP-bound forms of Cdc42 and, more weakly, to Rac, but genetic interactions further suggested that, *in vivo*, Frl is activated by Cdc42 [30]. Since suppression of either Cdc42 or Rac2 function phenocopies Frl/FMNL depletion in lamellocytes, we next analyzed the subcellular localization of Rac2 and Frl/FMNL. We recently established a new GFP-based sensor for active Rac1, Rac2 and Cdc42 termed MBT-GFP [38]. Different from a more evenly distributed Rac2-GFP protein trap (S1K Fig) this sensor marked distinct regions at the lamellocyte cell cortex (Fig 4A). Compared to MBT-GFP, anti-Frl/FMNL immunostainings revealed a more prominent localization of endogenous Frl/FMNL at the actin-enriched cell cortex in lamellocytes as well as in plasmatocytes, suggesting a conserved role in actin-based cortical dynamics (Fig 4B, arrowheads; Costes colocalization coefficient Frl/FMNL/F-actin: 0.6; [39]). *Tep4*-Gal4 driven RNAi-mediated depletion of Frl/FMN in lamellocytes but not plasmatocytes confirmed the antibody specificity (Fig 4C, 4D). Cortical immunostaining was completely lost in lamellocytes depleted for Frl/FMNL but not in plasmatocytes (Fig 4D, arrows). Interestingly, we found that both Cdc42 and Rac2 RNAi-mediated knockdown significantly reduced the localization Frl/FMNL at the cell cortex (Fig 4E, 4F). Quantitative analysis further revealed significant differences in the cortical Frl/FMNL intensity between Cdc42 and Rac2 knockdown (Fig 4G). Thus, the localization of Frl/FMNL depends on both Rho GTPases.

### Depletion of Frl/FMNL and Rac2 caused lamellocyte encapsulation defects after wasp infestation

**Fig 5:**
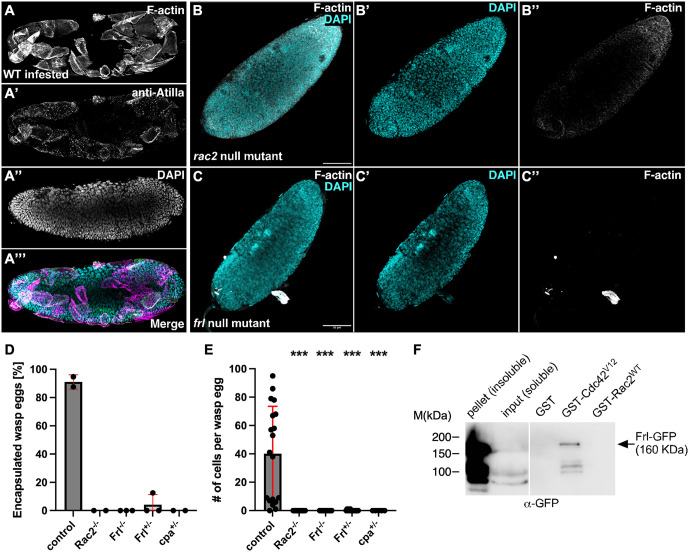
Both Rac2 and Frl/FMNL are required for attachment of lamellocytes to parasitic wasp eggs. **(A)** Confocal fluorescence microscopy image of a parasitic wasp egg recovered from a wildtype *Drosophila* larva. Note the larva number of attached cells positive for the lamellocyte marker Atilla (green). F-actin is stained with phalloidin (magenta) and nuclei with DAPI (cyan). Representative images from 3 independent biological replicates, 10 animals each. **(B-B”)** Parasitic wasp eggs recovered from *rac2* and **(C-C”)** *frl* null mutant larvae at the same timepoint after infestation. F-actin is stained with phalloidin (grey) and nuclei with DAPI (cyan). Scale bars: 50 µm. Representative images from 3 independent biological replicates, 10 animals each. **(D-E)** Quantification of cell attachment to parasitic wasp eggs. **(D)** *rac2*, *frl* and *cpa* mutants all show a drastic reduction in the amount of encapsulated wasp eggs compared to the control, two (control, Rac2^-/-^ and Cpa^+/-^) or three (Frl^-/-^ and Frl^+/-^) independent experiments (biological replicates from separate vials). **(E)** *rac2*, *frl* and *cpa* mutants all show a reduction in the number of cells attached to every wasp egg compared to the control. Same wasp eggs as in **(D)**, ***: p < 0.001. **(F)** Western Blot showing that most of Frl-GFP protein is found in the insoluble pellet fraction. Frl-GFP binds GST-Cdc42 but not GST-Rac2.

Given the prominent similar changes in the actin cytoskeleton and cell shape of lamellocytes depleted for Rac2, Cd42 and its known effector Frl/FMNL, we further analyzed possible encapsulation defects of a homozygous viable *frl/fmnl* protein null allele (*frl*^59^) as previously described [29,31]. Wild type larvae, homozygous *rac2* and *frl*^59^ mutant L1 instar larvae were parasitized by the low-virulent *L. boulardi* wasp strain G486 which hyperparsitizes larvae simplifying the imaging of lamellocyte-egg interactions [40]. As most of the wasp larvae hatched 30–32 h after infection [12] we isolated wasp eggs/larvae 24 hours after wasp infestation from L2 larvae and stained lamellocytes with the lamellocyte-specific anti-Atilla antibody L1. Isolated wasp eggs/larvae (visualized by DAPI staining) were partially encapsulated by numerous wild type lamellocytes, which had spread around the developing wasp eggs/larvae visualized by anti-Atilla and phalloidin staining (Fig 5A). By contrast, *rac2* mutants and *frl*^59^ mutants completely failed to encapsulate wasp eggs following larval parasitization (Fig 5B, 5C, quantification in Fig 5D, 5E). *frl*^59^ mutant lamellocytes failed to spread around the wasp egg/larvae similar to what was previously described for *cdc42* and *rac2* mutants [41,42]. As a consequence, only “naked” developing wasp larvae marked by strong nuclear DAPI staining could be observed from parasitized mutants, even in parasitized heterozygous *frl*^59^ mutants (Fig 5B, 5C, quantification in Fig 4E). From this, we conclude that both Rac2 and Frl/FMNL are necessary for proper capsule formation in response to parasitization. As a result, the morphologically-altered lamellocytes were unable to layer around the parasitoid eggs. Thus, these data suggest that proper lamellocyte spreading on the wasp egg requires Frl/FMNL-driven cortical actin reorganization downstream of both Rac2 and Cdc42. In GST pull down experiments we only confirmed a significant interaction between constitutively active Cdc42^V12^ and Frl/FMNL, which was expressed with a GFP tag in S2R+ cell culture (Fig 5F). However, the majority of this GFP-tagged Frl/FMNL protein was found in the insoluble lysate fraction despite increasing detergent concentrations up to 2%. We must also stress that we could not check the binding efficiency of constitutively active Rac2^V12^ for technical reason. Thus, it cannot be ruled out that weaker interactions between Frl/FMNL and Rac2 - which have already been demonstrated *in vitro* for human FMNL2 [43] - remain undetected here.

We finally employed AlphaFold3 to predict the structures of Frl/FMNL protein complexes with Cdc42 and Rac2 [44]. The structure of D*rosophila* Frl/FMNL fully resembled the known crystal structure of human FMNL2 (S2B and S2C Figs; compare S9 and S10 Movies). AlphaFold 3 indeed predicted that Frl/FMNL is able to directly bind Cdc42 but not Rac2. Interestingly, it also predicted Cdc42/Rac2 heterodimers that nicely fit into the FH3 binding pocket, both for *Drosophila* Frl/FMNL and human FMNL2 (S2B’ and S2C’ Fig; S9 and S10 Movies). Thus, such Cdc42/Rac2 heterodimers might act as an additional regulatory mechanism, differentially localizing Frl/FMNL complexes or differentially regulating intrinsic GTPase activity as previously shown for Cdc42 and Rac1 homodimers [45].

## Discussion

Previous studies have focused on mechanisms controlling *Drosophila* hematopoiesis, such as how multiple signaling pathways regulate the coordinated communication between the hematopoietic niche and blood progenitors under different physiological conditions [2,46,47]. The differentiation of lamellocytes, specialized immune cells crucial for fighting parasitic wasps, is therefore well characterized. However, our cellular understanding of this interesting cell type, its function, and regulation of its remarkable cytoskeleton is very limited. Findings on cell morphology and function of lamellocytes are based on pioneering studies conducted in the 1980s and 1990s [10, 16]. In this study, we revisited basic questions on their cell cytoskeleton and cell morphology using high-resolution imaging combined with a lamellocyte-specific candidate RNAi approach to identify key regulators of the lamellocyte actin cytoskeleton.

In some aspects, lamellocytes resemble the platelets in our bodies. Similar to platelets in our bloodstream, disc-shaped lamellocytes are non-migratory cells which passively circulate in the hemolymph. The dense actin filament network determines the rigid morphology of lamellocytes, which enables passive circulation with the hemolymph flow. When exposed to adhesive structures lamellocytes also undergo a dramatic reorganization of their cortical actin cytoskeleton. The reorganization of the lamellocyte actin cortex is accompanied by a significant change in cell elasticity, which we measured by using atomic force microscopy (AFM) for the first time. Once attached, lamellocytes become significantly softer while the cortical actin cytoskeleton reorganizes and drives lamellipodia-like protrusions to spread and to encapsulate the parasitoid wasp egg. Lamellocytes also express high levels of βPS-integrin, which might mediate a strong adhesion to matrix proteins on the parasitoid wasp eggs. Supporting this notion, the ability of lamellocytes to encapsulate has been shown to be disrupted in a βPS-integrin loss of function mutant [48]. However, it still remains unclear how lamellocytes adhere to the wasp egg surface. Previous studies suggested that a multilayered capsule is formed surrounding the wasp egg starting with an inner layer of plasmatocytes that recognize directly the wasp egg followed by newly differentiated lamellocytes interacting with the plasmatocyte layer in capsule formation [13,49]. We found no evidence for such a distinct plasmatocyte layer in capsule formation, both plasmatocytes and lamellocytes were found simultaneously on the wasp eggs isolated from infested L2 fly larvae. However, we indeed found dividing intermediates expressing both cell-lineage markers attached to a wasp egg as previously reported [12].

In mammalian cells, the binding of integrins to the extracellular matrix triggers a robust, intracellular signaling cascade that leads to the activation of Rac GTPases mediating cell spreading [50]. The *Drosophila* genome encodes three Rac GTPases: Rac1, Rac2 and Mig-2-like (Mtl) with overlapping functions in axon guidance in both the CNS and visual system [51]. Unlike in CNS development, Rac2 function is not redundant and is necessary for the encapsulation response of lamellocytes during the cellular immune response [41]. Both, Rac1 and Rac2 are master regulators of the actin cytoskeleton and key drivers of canonical lamellipodia, sheet-like membrane protrusions generated by WRC-Arp2/3-branched actin nucleation, which is essential for efficient mesenchymal cell migration [52,53]. However, cells lacking the WRC are still able to generate lamellipodia-like structures (LLS) driven by formins of the FMNL subfamily [35,36].

Interestingly, these lamellipodia-like structures (LLS) still require the presence of any of the three mammalian Rac GTPases and the related GTPase Cdc42 [35,37]. LLS are smaller in dimension and less efficient for effective cell migration compared to canonical WRC-dependent lamellipodia where Rac alone can trigger the formation of more efficient lamellipodia through WRC activation and Arp2/3 complex-dependent actin assembly [35]. Interestingly, WRC-Arp2/3-branched actin nucleation has no apparent function in the formation of lamellipodia-like protrusions of lamellocytes when spread on adhesive surfaces. Genetic or pharmacological inhibition of the Arp2/3 complex or its universal activator WAVE neither significantly affects the shape nor the overall actin cytoskeleton organization of lamellocytes. Instead, we found that the only ortholog of the FMNL subfamily in *Drosophila*, Frl/FMNL, as well as the GTPases Cdc42 and Rac2 are required for lamellocyte shape and proper spreading. Unlike plasmatocytes, non-migratory lamellocytes only passively circulate in the hemolymph, thus do not require an efficient cell migration mode. Instead, lamellocytes require Frl/FMNL-driven protrusion downstream of Rac2 and Cdc42 for efficient spreading on the wasp egg. Accordingly, we found that *frl/fmnl* mutant lamellocytes completely failed to spread around the wasp egg/larvae similar to what was previously described for *cdc42* and *rac2* mutants [41,42]. Previous studies also demonstrated that formins such as FMNL2 together with capping protein functionally coregulate filament barbed-end assembly to drive protrusion dynamics [54,55]. In support of this notion, depletion of Capping protein (Cpa) also resulted in strong lamellocyte encapsulation defects as observed for *frl/fmnl* mutants (see quantification in Fig 5D, 5E). Unlike *frl/fmnl2* knockdown, depletion of *cpa* did not affect lamellocyte shape but rather induced a massive membrane ruffling at the dorsal surface of lamellocytes. In wild type lamellocytes, such intense membrane ruffles were only observed during initial cell spreading but not in fully spread lamellocytes. The function of these membrane ruffles during lamellocyte spreading remains unknown.

The actin cytoskeleton is a prime target of bacterial, viral pathogens and eukaryotic parasites and inactivation of host Rho GTPases is a widespread strategy employed by pathogens to impair cellular functions and avoid immune defenses [56–59]. Rho GAPs have been identified as virulence factors in both bacteria and eukaryotic parasites [57,60,61].

A striking example is the venom protein LbGAP of *Leptopilina boulardi* which only consists of a signal peptide required for secretion and a RhoGAP domain that specifically targets *Drosophila* Rac1 and Rac2 to protect parasitoid eggs from the immune response of lamellocytes [57]. Parasitoid wasps have evolved multiple venom virulence proteins that allow them to overcome the host immune response [62,63]. Some of these venom proteins also specifically target the lamellocyte microtubule cytoskeleton such as Lamellolysin, a virulence factor from *Leptopilina heterotoma*, which specifically targets and destroys lamellocytes by disrupting their microtubule cytoskeleton [16,64,65]. To our knowledge, the exact nature of Lamellolysin has been never identified but these studies highlight key roles of the actin and microtubule cytoskeleton in the cellular immune defense as well as the remarkable molecular evolution and functional convergence of venom proteins in host-pathogen interactions.

## Materials and methods

### Drosophila genetics

Fly husbandry and crossing were carried out according to standard methods. Crosses were maintained at 25°C. The following fly lines were used: UAS-*luciferase* RNAi (Bloomington *Drosophila* Stock Center (BDSC) 31603), UAS-*arp3* RNAi (Vienna Drosophila Resource Center (VDRC) 35258), UAS-*wave* RNAi (National Institute of Genetics (NIG) 4636), UAS-*cpa* RNAi (VDRC 100773), UAS-*rac2* RNAi (NIG 8556R-3), UAS-*cdc42* RNAi (VDRC 100794), UAS-*frl* RNAi (VDRC 34412), UAS-*rac1* RNAi (BDSC 34910), *atilla*-Gal4 (BDSC 23540), *msnF9*-Gal4 [66], *rac2*^Δ^,*ry*^506^ (BDSC 6675), *cpa*^*KO*^*/CyO* (BDSC 93756), *hop*^*Tum-l*^/FM7c (BDSC 8492), *frl*^59^/TM6,ubi-GFP (a gift from J. Mihály, University of Szeged), OregonR (a gift from C. Klämbt, University of Münster), UAS-MBT-CRIB-GFP [67]. *Tep4*-Gal4/SM6a (BDSC 76750) and UAS-Lifeact-GFP/CyO;TM2/TM6b were recombined to yield *tep4*-Gal4,UAS-Lifeact-GFP/CyO.

For wasp infestation, 10–15 female and three to five male flies were transferred to a fresh food vial and placed at 25°C for 48 h. Flies were then removed and five to eight females and three to five male parasitoid wasps of the species *Leptopilina boulardi*, strain G486 (a gift from Jean-Luc Gatti, Institut Sophia Agrobiotech), were added to parasitize the fly larvae at 25°C. After 2 days, wasps were removed before dissecting larvae as described below. Before dissection, parasitization was confirmed by the presence of melanized injection sites.

### Bioinformatic analysis

We used a published single-cell transcriptomics dataset of hemocytes from wasp-infested L3 larvae [20] to assess differences in gene expression between plasmatocytes and lamellocytes. The published gene matrix was quality-controlled, keeping only genes with at least 200 reads from at least 3 cells. UMAP coordinates were calculated and cells clustered with 10 dimensions at a resolution factor of 0.7. Clusters were validated based on the differential expression of a set of well-established marker genes. For simplicity, clusters of plasmatocyte and lamellocyte identity respectively, were combined to result in a clustering by cell type.

Protein structure and interaction predictions were calculated and visualized using AlphaFold3 [44] on the AlphaFold server (https://alphafoldserver.com/) using protein sequences for *Drosophila* Frl (UniProt accession Q9VUC6), Rac2 (UniProt accession P48554), Cdc42 (UniProt accession P40793) and a consensus sequence for human FMNL2 (UniProt accession Q96PY5).

### Immunohistochemistry

Larval hemocytes were isolated as described previously [25]. In short, wandering L3 larvae were collected and washed in 1 × PBS. The larvae were then transferred to 1 × Schneider’s *Drosophila* medium (Gibco) supplemented with 10% fetal bovine serum, 50 units/ml penicillin and 50 µg/ml streptomycin and opened using tweezers to release the hemolymph. Cells were spread on glass coverslips, previously coated for 30 min with Concanavalin A (0.5 mg/ml, Sigma-Aldrich), for 1 h at 25°C. For inhibitor treatments, colchicine (5µg/ml, Calbiochem) in water, Latrunculin A (1µmol/l, Cayman Chemical) in dimethyl sulfoxide (DMSO, Carl Roth), CK-666 (200µmol/l, Merck) in DMSO or equivalent amounts of water or DMSO as controls were added to the medium during this hour. The supernatant was removed and adherent cells were subsequently fixed for 15 min with 4% paraformaldehyde in 1 × PBS at room temperature (RT). Cells were briefly rinsed with 1 × PBS + 0.1% Triton X-100 immediately followed by three washing steps with 1 × PBS. If no antibody staining was performed, the treatment with the PBS-Triton solution was omitted. Cells were stained with primary antibody for 2 hours at RT and secondary antibody, fluorescently labelled Phalloidin and DAPI for 1 hour at RT in a humidified dark chamber. Stained cells were mounted in Mowiol 4–88 (Carl Roth). The following primary antibodies were used: Mouse anti-Atilla (1:100; [19], mouse anti-βtubulin (1:100, E7 from Developmental Studies Hybridoma Bank), guinea-pig anti-WAVE (1:100; [67] and rat anti-Frl (1:200, a gift from J. Mihály, [31]. The following secondary antibodies were used: polyclonal Alexa Fluor-647-conjugated goat-anti-mouse (1:1000, A21236, Invitrogen) and polyclonal Alexa Fluor-647-conjugated goat-anti-rat (1:1000, A21247, Invitrogen). F-actin was stained using Phalloidin-Alexa Fluor 568 (1:100, A12380, Invitrogen) and nuclei with DAPI (1 µg/ml, 62248, Thermo Fisher Scientific).

### Preparation of encapsulated parasitoid wasp eggs from fly larvae

Flies were kept in a cage with apple juice agar and some fresh yeast paste at the bottom for 24 hours at 25°C. After the flies were removed, the eggs were incubated for 24 hours at 25°C, until the emergence of L1 larvae. 8–10 female and 3–5 male *Leptopilina boulardi* G486 parasitoid wasps were added for 4 hours at 25°C. Wasp eggs were dissected from L2 larvae 24 hours after wasp infestation in dissection medium (see isolation of hemocytes). For live imaging, eggs were transferred to imaging medium (see live-cell imaging) in an 8 well imaging chamber (Lab-Tek). For fixation, eggs were collected in a drop of dissection medium (see above) on a silane prep slide (hydrophobic glass slide, Sigma Aldrich #S4651-72EA). After careful removal of the medium, eggs were fixed with 4% paraformaldehyde for 30 min at RT, then stained with Phalloidin-Alexa Fluor 568 (1:100, Invitrogen) and DAPI (1:1000, Thermo Fisher Scientific) for 1 hour at RT. For quantifications, wasp eggs with at least one attached cell were scored as “encapsulated”.

### Live-cell imaging of lamellocytes

Larvae were collected as for fixed samples but dissected in imaging medium (1 × Schneider’s *Drosophila* medium (Gibco) supplemented with 10% fetal bovine serum, 50 units/ml penicillin, 50 µg/ml streptomycin, 20 µg/ml gentamycin, 10nmol/l *N*-phenylthiourea and 10 mg/ml insulin) and spread on Concanavalin A-treated 8 well imaging chambers (Lab-Tek). Long-term spinning disk time-lapse movies were generated by taking images every 60 s for 8 hours.

For *in vivo* imaging of lamellocytes, larvae were briefly chilled on ice, then placed ventral side down in a large glass-bottom dish (WillCo) between two stacks of three layers of tape as spacers. A cover slip with double-sided tape was then placed on the spacers, exerting gentle pressure on the larva to keep it in place. Time-lapse movies were generated by taking images at maximum acquisition speed (516 ms interval) for 10 minutes.

### Image acquisition and microscopy

Confocal fluorescent images were taken with a Leica TCS SP8 with an HC PL APO CS2 63 × /1.4 oil objective and Leica Application Suite X (LasX, Version 3.5.2.18963).

*Ex vivo* time-lapse imaging of lamellocytes was performed using a Zeiss CellObserver Z.1 with a Yokogawa CSU-X1 spinning disk scanning unit and an Axiocam MRm CCD camera (6.45 µm × 6.45 µm) and ZenBlue 2.6 software. Super-resolution images were taken with a Zeiss LSM 980 Airyscan 2 and ZenBlack software.

### Image analysis

For measurements of cell area, circularity or fluorescent signal intensity, cells were randomly chosen. Thresholds were set in ImageJ (Version 1.54p) based on the phalloidin staining and the respective values measured. Circularity is given as 4π(cell area/cell perimeter^2^) and describes the similarity of an object to a perfect circle. Fluorescent signal intensity was normalized to the background (cell-free area of the same image). For calculating the cortex:cell body-ratio, intensity was first measured as described (see also S2 Fig). The selection was then converted to the cell perimeter using the command *area to line* to measure fluorescence intensity along the cell outline.

### Cell culture and cell transfection

*Drosophila* hemocyte-like S2R+ cells [68] were cultured in Schneider’s medium as previously described [69]. Cell transfection was performed using Fugene (Promega) as described in [69]. The transfected plasmid was pUAST attB GFP Frl. The pUAST attB GFP Frl construct was created by Gateway recombination cloning (Invitrogen): The full length Frl gene from pENTR1A Frl (entry vector, a kind gift from J. Mihály) was cloned into pUAST attB GFP rfa (destination vector) by recombination using LR clonase enzyme mix (Invitrogen).

### Recombinant protein expression and GST pulldown assay

Expression of GST-tagged Cdc42^V12^ (cloned into pGEX 6P1; Addgene) and Rac2^WT^ (cloned into pGEX 2T; Addgene, a gift from J. Grosshans, Philipps University of Marburg) was induced in ArcticExpress cells (Agilent Technologies) with 1mM IPTG for 24 hours at 10°C. Cells were lysed by sonication in GST lysis buffer (2 mM MgCl2, 2 mM DTT, 10% (vol/vol) glycerol in 1 × PBS). GST pulldowns were performed as previously described [67]. Recombinant GST, GST-Rac2^WT^ and GST-Cdc42^V12^ was bound to 30µl Glutathione Sepharose 4B beads (Merck, Cytiva). *Drosophila* S2R+ cells transfected with pUAST attB GFP Frl were harvested 72h post transfection and lysed in TLB buffer (50 mM Tris-HCL pH 7.4, 150 mM NaCl, 1.5 mM MgCl_2_, 4 mM EDTA, 10% [vol/vol] glycerol, 2% [vol/vol] Triton X-100, 1 mM DTT) by vortexing for one hour at room temperature. The cell lysates were incubated over night at 4°C with GST, GST-Rac2 and GST-Cdc42^V12^ before beads were washed and prepared for SDS PAGE and Western blot.

### SDS PAGE and Western blot analysis

4 × SDS sample buffer was added to S2R+ cell lysates and Glutathione Sepharose beads were resuspended with 1 × SDS sample buffer and incubated for 10 min at 95°C. Samples were then separated by SDS PAGE and analyzed by Western Blot. The following antibodies were used: mouse anti-GFP (1:1,000 dilution; ClonTech JL-8 #632381, incubation over night at 4°C), goat anti-mouse IgG HRP (1:10,000 dilution; Jackson ImmunoResearch #115-035-003, incubation 2 hours at room temperature). Bands were visualized using a chemiluminescent HRP substrate (SuperSignal West Dura, ThermoFisher) according to the manufacturer’s specifications.

### Elasticity measurement

Elasticity measurements were conducted using a Bioscope Resolve atomic force microscope (Bruker; Santa Barbara, USA) operated in closed-loop contact mode with a ramp size of 4 µm, max. loading force of 1 nN and a tip velocity of 1 µm/s. Freshly isolated cells were placed in Concanavalin A coated 35 × 10 mm dishes (CytoOne; Starlab) and allowed to settle for 30 minutes before measurement. AFM measurements were performed in dissection medium at room temperature, using an active anti-vibration platform (Halcyonics_i4; Accurion) to minimize background noise. Drift-compensated spherical probes (MLCT-SPH-5UM-DC; Bruker AFM Probes, Camarillo, USA) with tip radius of 4.9 µm were employed to assess the mechanical properties of the cell. The cantilever, pre-calibrated by the manufacturer, had a spring constant of 0.017 N/m and deflection sensitivity was calibrated before testing according to the standardized nanomechanical AFM procedure [70]. The analysis of the force–indentation curves was performed with PUNIAS (Protein Unfolding and Nano-Indentation Analysis Software; http://punias.free.fr) using the linearized Hertz model to determine cell elasticity as Young’s modulus (E) [71].

### PFQNM (Peak Force Quantitative Nanomechanical Mapping)

AFM imaging of glutaraldehyde fixed cells was performed in PBS at room temperature in PeakForce Tapping mode [72] using a BioScope Resolve AFM (Bruker Nano Surfaces, Santa Barbara, CA, USA). A PeakForce QNM‐Live Cell (PFQNM‐LC-V2) probe (Bruker AFM Probes, Camarillo, CA, USA) (tip radius 70 nm) was used to image the cell surface. The cantilever, pre-calibrated by the manufacturer, had a spring constant of 0.148 N/m and deflection sensitivity was calibrated before testing according to the standardized nanomechanical AFM procedure [70]. Images were taken with a PeakForce setpoint of 500pN at 256 × 256 pixels with a scan rate of 0.3 Hz. PeakForce Tapping frequency and amplitude was set to 1 kHz and 300 nm respectively. Height sensor and PeakForce error signals was used to display the cell surface image using MountainsSPIP version 10.0.10656 (Digital Surf, Besançon, France).

### Statistical analysis

All statistical tests were performed in GraphPad Prism (Version 10.4.0). The respective tests are indicated in the figure legends but generally, Mann-Whitney tests were used for comparisons of two groups and One-way ANOVA with Kruskal-Wallis test was used when comparing more than two experimental groups. Unless otherwise indicated, cells were randomly selected for quantification.

## Supporting information

S1 Fig (A) Table of candidate genes in the RNAi knockdown screen. Genes, whose knockdown resulted in obvious changes in lamellocyte morphology are marked in green. Genes, where knockdown was tested but did not result in obvious morphology changes are marked in grey. For every gene, a representative number of cells from two independent experiments were screened for atypical morphology. **(B-B’)** The same RNAi construct that did not show a morphological phenotype in lamellocytes (see Fig 3E) causes long cytoskeletal protrusions in plasmatocytes under the control of *hmlΔ*-Gal4. Expression of the Gal4 driver is marked by GFP. Lamellocytes are rare but can occur in uninfected animals and are negative for *hmlΔ*-Gal4. F-actin is stained with phalloidin (grey) and nuclei with DAPI (blue). Scale bar: 10 µm. Representative images from 3 independent biological replicates, 10 animals each. **(C-D’)** Immunofluorescence staining showing efficient knockdown of WAVE in lamellocytes. **(C-C’)** In the control condition, lamellocytes strongly express WAVE as shown by specific antibody staining (magenta). **(D-D’)** This staining is largely lost upon knockdown of WAVE. F-actin is stained with phalloidin (grey) and nuclei with DAPI (blue). Scale bars: 10 µm. Representative images from 3 independent biological replicates, 10 animals each. **(E-F)** Lamellocyte morphology is not affected by treatment with the Arp2/3 inhibitor CK-666. **(E)** Lamellocytes (marked by antibody staining against Atilla (magenta)) and plasmatocytes from *hop*^*Tum-l*^ larvae after treatment with DMSO (control). **(F)** Lamellocytes and plasmatocytes from *hop*^*Tum-l*^ larvae after treatment with CK-666 (200µmol/l). Note the long cytoskeletal extensions in plasmatocytes (arrows) while lamellocytes are unaffected (asterisks). F-actin is stained with phalloidin (grey) and nuclei with DAPI (blue). Scale bars: 10 µm. Representative images from 3 independent biological replicates, 10 animals each. **(G)** Quantification showing that expression level of the *tep4*-Gal4 driver (measured by the intensity of Lifeact-GFP) is unchanged by knockdown of *rac2*, *cdc42* and *frl*. N = 12 (control), N = 9 (*rac2* RNAi), N = 24 (*cdc42* RNAi) and N = 26 cells (*frl* RNAi; technical replicates from all experiments) cells from 2 independent biological replicates, 10 animals each. One-way ANOVA with Kruskal-Wallis test. All comparisons are non-significant (p > 0.05). **(H-J)** Confocal microscopy images of lamellocytes after knockdown of **(H)** the actin nucleator *spir*, the formins **(I)** *capu* and **(J)** *fhos*. F-actin is visualized with phalloidin (grey) and nuclei with DAPI (blue). Scale bars: 10 µm. Representative images from 3 independent biological replicates, 10 animals each. **(K-K”)** Lamellocyte with endogenously GFP-labelled Rac2 **(K”)**, showing an even localization of Rac2 in the cell. F-actin is stained with phalloidin (grey) and nuclei with DAPI (blue). Scale bar: 10 µm. Representative images from 2 independent biological replicates, 10 animals each.
(TIF)

S2 Fig (A) Schematic showing how signals at the cortex, in the cell body (cytoplasm) and the cortex-body ratio were quantified. **(B)** *In silico* AlphaFold 3 prediction of *Drosophila* Frl/FMNL structure. The diaphanous autoregulatory domain (DAD) is marked in red, FH2 domain is marked in blue and GTPase binding domain is marked in purple. **(B’)** *In silico* AlphaFold 3 interaction prediction of *Drosophila* Frl/FMNL (same color code as in G), Cdc42 (orange) and Rac2 (green). Predicted template modelling score (pTM) = 0.42. **(C)** *In silico* AlphaFold 3 structural prediction of human FMNL2 (same color code as for the *Drosophila* homolog in G). **(C’)** *In silico* AlphaFold 3 interaction prediction between human FMNL2 (same color code as for the *Drosophila* homolog in G), Cdc42 (orange) and Rac2 (green). pTM = 0.4.
(TIF)

S1 Raw Gel Original western blot image shown in Fig 5F. The middle control lanes (marked in red), in which GST proteins were coupled to beads without cell lysate, were omitted from Fig 5F for clarity.
(TIF)

S1 Movie *Ex vivo* spinning-disk confocal timelapse movie of larval lamellocytes from wasp-infested L3 instar larvae expressing a Lifeact-GFP transgene under the control of the Tep4-Gal4 driver, plated on glass surfaces. Note the very rigid, curved, disc-shaped form of the lamellocytes compared to smaller motile macrophage-like plasmatocytes with highly dynamic protrusions. Representative results from 10 independent experiments/vials each isolated from 5-10 larvae.
(MP4)

S2 Movie Timelapse microscopy movie of a *hop*^*Tum-l*^ mutant larva with circulating lamellocytes expressing lifeact-GFP under control of the Tep4-Gal4 driver. Note the discoidal cells passively following the hemolymph flow. Scale bar: 200 µm. Representative results from 5 independent experiments/vials each isolated from 5-10 larvae.
(MP4)

S3 Movie Long-term-spinning-disk confocal timelapse movie of larval lamellocytes isolated from *hop*^*Tum-l*^ mutants expressing a Lifeact-GFP transgene under the control of the Tep4-Gal4 driver, plated on concanavalin A-coated surfaces. Scale bar: 10 µm. Representative results from 10 independent experiments/vials each isolated from 5-10 larvae.
(MP4)

S4 Movie 3D Imaris reconstruction movie of a larval lamellocyte isolated from *hop*^*Tum-l*^ mutants expressing a Lifeact-GFP transgene under the control of the Tep4-Gal4 driver, plated on Concanavalin A-coated surfaces. Scale bar: 10 µm. Representative results from 5 independent experiments/vials each isolated from 5-10 larvae.
(MP4)

S5 Movie Spinning disk microscopy timelapse movie of a parasitoid wasp larva isolated from infested L2 fly larvae expressing the dual reporter msn-Cherry/eater-GFP-NLS. Plasmatocytes are marked by *eater*-GFP-NLS, while lamellocytes express *msn*-mCherry. Frequently, lamellocytes are positive for both markers. Yellow arrow marks a double-positive cell which starts cell division. Scale bar: 20 µm. Representative results from 10 independent experiments/vials.
(MP4)

S6 Movie Spinning disk microscopy timelapse movie of a parasitoid wasp egg/larva isolated from infested L2 fly larvae expressing the Lifeact-GFP reporter under the control of *Tep4*-Gal4. Lamellocytes show numerous F-actin rich lamellipodia-like membrane protrusions and ruffles. Expression levels of *Tep4*-Gal4 increase over the course of several hours of spreading. Scale bar: 10 µm. Representative results from 10 independent experiments/vials.
(MP4)

S7 Movie Long-term-spinning-disk confocal timelapse movie of larval lamellocytes isolated from *hop*^*Tum-l*^ mutants expressing a Lifeact-GFP transgene under the control of the *Tep4*-Gal4 driver, plated on concanavalin A-coated surfaces. **(Left)** Lamellocytes were treated with colchicine and **(Right)** with Latrunculin A. Scale bars: 10 µm. Representative results from 5 independent experiments/vials.
(MP4)

S8 Movie *Ex vivo* confocal microscopy timelapse movie of larval lamellocytes depleted for capping protein A (Cpa). F-actin dynamics are visualized by Lifeact-GFP. Note intense membrane ruffles and dynamic ring-like F-actin accumulation around vesicular structures. Scale bar: 10 µm. Representative results from 10 independent experiments/vials.
(MP4)

S9 Movie *In silico* AlphaFold 3 structure and interaction prediction of *Drosophila* Frl/FMNL with Rac2 (green) and Cdc42 (orange). (MP4)

S10 Movie *In silico* AlphaFold 3 structure and interaction prediction of human FMNL2 with Rac2 (green) and Cdc42 (orange). (MP4)

## References

[1] Dupré L, Castanon I, Boztug K. Immune-related actinopathies at the cross-road of immunodeficiency, autoimmunity and autoinflammation. Nat Rev Immunol. 2026;26(2):89–111. doi: 10.1038/s41577-025-01214-w 40931027

[2] Banerjee U, Girard JR, Goins LM, Spratford CM. Drosophila as a genetic model for hematopoiesis. Genetics. 2019;211(2):367–417. doi: 10.1534/genetics.118.300223 30733377 PMC6366919

[3] Evans CJ, Hartenstein V, Banerjee U. Thicker than blood: conserved mechanisms in Drosophila and vertebrate hematopoiesis. Dev Cell. 2003;5(5):673–90. doi: 10.1016/s1534-5807(03)00335-6 14602069

[4] Evans IR, Wood W. Drosophila blood cell chemotaxis. Curr Opin Cell Biol. 2014;30:1–8. doi: 10.1016/j.ceb.2014.04.002 24799191 PMC4194352

[5] Lemaitre B, Hoffmann J. The host defense of Drosophila melanogaster. Annu Rev Immunol. 2007;25:697–743. 17201680 10.1146/annurev.immunol.25.022106.141615

[6] Gold KS, Brückner K. Drosophila as a model for the two myeloid blood cell systems in vertebrates. Exp Hematol. 2014;42(8):717–27. doi: 10.1016/j.exphem.2014.06.002 24946019 PMC5013032

[7] Honti V, Kurucz E, Csordás G, Laurinyecz B, Márkus R, Andó I. In vivo detection of lamellocytes in Drosophila melanogaster. Immunol Lett. 2009;126(1–2):83–4. doi: 10.1016/j.imlet.2009.08.004 19695290

[8] Lanot R, Zachary D, Holder F, Meister M. Postembryonic hematopoiesis in Drosophila. Dev Biol. 2001;230(2):243–57. doi: 10.1006/dbio.2000.0123 11161576

[9] Carton Y, Boulétreau M. Encapsulation ability of Drosophila melanogaster: a genetic analysis. Dev Comp Immunol. 1985;9(2):211–9. doi: 10.1016/0145-305x(85)90112-0 3926550

[10] Rizki TM, Rizki RM. Parasitoid-induced cellular immune deficiency in Drosophila. Ann N Y Acad Sci. 1994;712:178–94. doi: 10.1111/j.1749-6632.1994.tb33572.x 7910721

[11] Strand MR, Pech LL. Immunological basis for compatibility in parasitoid-host relationships. Annu Rev Entomol. 1995;40:31–56. doi: 10.1146/annurev.en.40.010195.000335 7810989

[12] Anderl I, Vesala L, Ihalainen TO, Vanha-Aho L-M, Andó I, Rämet M, et al. Transdifferentiation and proliferation in two distinct hemocyte lineages in Drosophila melanogaster Larvae after Wasp Infection. PLoS Pathog. 2016;12(7):e1005746. doi: 10.1371/journal.ppat.1005746 27414410 PMC4945071

[13] Russo J, Dupas S, Frey F, Carton Y, Brehelin M. Insect immunity: early events in the encapsulation process of parasitoid (Leptopilina boulardi) eggs in resistant and susceptible strains of Drosophila. Parasitology. 1996;112 (Pt 1):135–42. doi: 10.1017/s0031182000065173 8587797

[14] Hultmark D, Andó I. Hematopoietic plasticity mapped in Drosophila and other insects. Elife. 2022;11:e78906. doi: 10.7554/eLife.78906 35920811 PMC9348853

[15] Wan B, Belghazi M, Lemauf S, Poirié M, Gatti J-L. Proteomics of purified lamellocytes from Drosophila melanogaster HopTum-l identifies new membrane proteins and networks involved in their functions. Insect Biochem Mol Biol. 2021;134:103584. doi: 10.1016/j.ibmb.2021.103584 34033897

[16] Rizki RM, Rizki TM. Selective destruction of a host blood cell type by a parasitoid wasp. Proc Natl Acad Sci U S A. 1984;81(19):6154–8. doi: 10.1073/pnas.81.19.6154 6435126 PMC391878

[17] Kontomaris SV, Malamou A, Stylianou A. Development of an accurate simplified approach for data processing in AFM indentation experiments. Micron. 2025;190:103782. 39799615 10.1016/j.micron.2024.103782

[18] Luo H, Hanratty WP, Dearolf CR. An amino acid substitution in the Drosophila hopTum-l Jak kinase causes leukemia-like hematopoietic defects. EMBO J. 1995;14(7):1412–20. doi: 10.1002/j.1460-2075.1995.tb07127.x 7729418 PMC398227

[19] Kurucz E, Váczi B, Márkus R, Laurinyecz B, Vilmos P, Zsámboki J, et al. Definition of Drosophila hemocyte subsets by cell-type specific antigens. Acta Biol Hung. 2007;58 Suppl:95–111. doi: 10.1556/ABiol.58.2007.Suppl.8 18297797

[20] Cattenoz PB, Sakr R, Pavlidaki A, Delaporte C, Riba A, Molina N, et al. Temporal specificity and heterogeneity of Drosophila immune cells. EMBO J. 2020;39:e104486. 10.15252/embj.2020104486 PMC7298292 32162708

[21] Cho B, Yoon S-H, Lee D, Koranteng F, Tattikota SG, Cha N, et al. Single-cell transcriptome maps of myeloid blood cell lineages in Drosophila. Nat Commun. 2020;11(1):4483. doi: 10.1038/s41467-020-18135-y 32900993 PMC7479620

[22] Fu Y, Huang X, Zhang P, van de Leemput J, Han Z. Single-cell RNA sequencing identifies novel cell types in Drosophila blood. J Genet Genomics. 2020;47(4):175–86. doi: 10.1016/j.jgg.2020.02.004 32487456 PMC7321924

[23] Hirschhäuser A, Molitor D, Salinas G, Großhans J, Rust K, Bogdan S. Single-cell transcriptomics identifies new blood cell populations in Drosophila released at the onset of metamorphosis. Development. 2023;150(18):dev201767. doi: 10.1242/dev.201767 37681301 PMC10560556

[24] Rohn JL, Sims D, Liu T, Fedorova M, Schöck F, Dopie J, et al. Comparative RNAi screening identifies a conserved core metazoan actinome by phenotype. J Cell Biol. 2011;194(5):789–805. doi: 10.1083/jcb.201103168 21893601 PMC3171124

[25] Rüder M, Nagel BM, Bogdan S. Analysis of cell shape and cell migration of Drosophila macrophages in vivo. Methods Mol Biol. 2018;1749:227–38. doi: 10.1007/978-1-4939-7701-7_17 29526001

[26] Sander M, Squarr AJ, Risse B, Jiang X, Bogdan S. Drosophila pupal macrophages--a versatile tool for combined ex vivo and in vivo imaging of actin dynamics at high resolution. Eur J Cell Biol. 2013;92(10–11):349–54. doi: 10.1016/j.ejcb.2013.09.003 24183239

[27] Hetrick B, Han MS, Helgeson LA, Nolen BJ. Small molecules CK-666 and CK-869 inhibit actin-related protein 2/3 complex by blocking an activating conformational change. Chem Biol. 2013;20(5):701-12. doi: 10.1016/j.chembiol.2013.03.019 23623350 PMC3684959

[28] Lawson CD, Ridley AJ. Rho GTPase signaling complexes in cell migration and invasion. J Cell Biol. 2018;217:447–57. 29233866 10.1083/jcb.201612069 PMC5800797

[29] Dehapiot B, Clément R, Alégot H, Gazsó-Gerhát G, Philippe J-M, Lecuit T. Assembly of a persistent apical actin network by the formin Frl/Fmnl tunes epithelial cell deformability. Nat Cell Biol. 2020;22(7):791–802. doi: 10.1038/s41556-020-0524-x 32483386

[30] Dollar G, Gombos R, Barnett AA, Sanchez Hernandez D, Maung SM, Mihaly J, et al. Unique and overlapping functions of Formins Frl and DAAM during ommatidial rotation and neuronal development in Drosophila. Genetics. 2016;202:1135–51. 26801180 10.1534/genetics.115.181438 PMC4788114

[31] Tóth K, Földi I, Mihály J. A comparative study of the role of Formins in Drosophila embryonic dorsal closure. Cells. 2022;11(9):1539. doi: 10.3390/cells11091539 35563844 PMC9102720

[32] Bear JE, Svitkina TM, Krause M, Schafer DA, Loureiro JJ, Strasser GA, et al. Antagonism between Ena/VASP proteins and actin filament capping regulates fibroblast motility. Cell. 2002;109(4):509–21. doi: 10.1016/s0092-8674(02)00731-6 12086607

[33] Mejillano MR, Kojima S, Applewhite DA, Gertler FB, Svitkina TM, Borisy GG. Lamellipodial versus filopodial mode of the actin nanomachinery: pivotal role of the filament barbed end. Cell. 2004;118(3):363–73. doi: 10.1016/j.cell.2004.07.019 15294161

[34] Yayoshi-Yamamoto S, Taniuchi I, Watanabe T. FRL, a novel formin-related protein, binds to Rac and regulates cell motility and survival of macrophages. Mol Cell Biol. 2000;20(18):6872–81. doi: 10.1128/MCB.20.18.6872-6881.2000 10958683 PMC86228

[35] Kage F, Döring H, Mietkowska M, Schaks M, Grüner F, Stahnke S, et al. Lamellipodia-like actin networks in cells lacking WAVE regulatory complex. J Cell Sci. 2022;135(15):jcs260364. doi: 10.1242/jcs.260364 35971979 PMC9511706

[36] Kage F, Steffen A, Ellinger A, Ranftler C, Gehre C, Brakebusch C, et al. FMNL2 and -3 regulate Golgi architecture and anterograde transport downstream of Cdc42. Sci Rep. 2017;7(1):9791. doi: 10.1038/s41598-017-09952-1 28852060 PMC5575334

[37] Kühn S, Erdmann C, Kage F, Block J, Schwenkmezger L, Steffen A, et al. The structure of FMNL2-Cdc42 yields insights into the mechanism of lamellipodia and filopodia formation. Nat Commun. 2015;6:7088. doi: 10.1038/ncomms8088 25963737 PMC4432619

[38] Rötte M, Höhne MY, Klug D, Ramlow K, Zedler C, Lehne F, et al. CYRI controls epidermal wound closure and cohesion of invasive border cell cluster in Drosophila. J Cell Biol. 2024;223(12). doi: 10.1083/jcb.202310153 39453414 PMC11519390

[39] Costes SV, Daelemans D, Cho EH, Dobbin Z, Pavlakis G, Lockett S. Automatic and quantitative measurement of protein-protein colocalization in live cells. Biophys J. 2004;86(6):3993–4003. doi: 10.1529/biophysj.103.038422 15189895 PMC1304300

[40] Sorrentino RP, Carton Y, Govind S. Cellular immune response to parasite infection in the Drosophila lymph gland is developmentally regulated. Dev Biol. 2002;243(1):65–80. doi: 10.1006/dbio.2001.0542 11846478

[41] Williams MJ, Ando I, Hultmark D. Drosophila melanogaster Rac2 is necessary for a proper cellular immune response. Genes Cells. 2005;10(8):813–23. doi: 10.1111/j.1365-2443.2005.00883.x 16098145

[42] Xavier MJ, Williams MJ. The Rho-family GTPase Rac1 regulates integrin localization in Drosophila immunosurveillance cells. PLoS One. 2011;6(5):e19504. doi: 10.1371/journal.pone.0019504 21603603 PMC3095607

[43] Block J, Breitsprecher D, Kühn S, Winterhoff M, Kage F, Geffers R, et al. FMNL2 drives actin-based protrusion and migration downstream of Cdc42. Curr Biol. 2012;22(11):1005–12. doi: 10.1016/j.cub.2012.03.064 22608513 PMC3765947

[44] Abramson J, Adler J, Dunger J, Evans R, Green T, Pritzel A, et al. Accurate structure prediction of biomolecular interactions with AlphaFold 3. Nature. 2024;630(8016):493–500. doi: 10.1038/s41586-024-07487-w 38718835 PMC11168924

[45] Zhang B, Zheng Y. Negative regulation of Rho family GTPases Cdc42 and Rac2 by homodimer formation. J Biol Chem. 1998;273(40):25728–33. doi: 10.1074/jbc.273.40.25728 9748241

[46] Morin-Poulard I, Tian Y, Vanzo N, Crozatier M. Drosophila as a model to study cellular communication between the hematopoietic niche and blood progenitors under homeostatic conditions and in response to an immune stress. Front Immunol. 2021;12:719349. doi: 10.3389/fimmu.2021.719349 34484226 PMC8415499

[47] Yu S, Luo F, Xu Y, Zhang Y, Jin LH. Drosophila innate immunity involves multiple signaling pathways and coordinated communication between different tissues. Front Immunol. 2022;13:905370. doi: 10.3389/fimmu.2022.905370 35911716 PMC9336466

[48] Irving P, Ubeda J-M, Doucet D, Troxler L, Lagueux M, Zachary D, et al. New insights into Drosophila larval haemocyte functions through genome-wide analysis. Cell Microbiol. 2005;7(3):335–50. doi: 10.1111/j.1462-5822.2004.00462.x 15679837

[49] Mortimer NT, Kacsoh BZ, Keebaugh ES, Schlenke TA. Mgat1-dependent N-glycosylation of membrane components primes Drosophila melanogaster blood cells for the cellular encapsulation response. PLoS Pathog. 2012;8(7):e1002819. doi: 10.1371/journal.ppat.1002819 22829770 PMC3400557

[50] Price LS, Leng J, Schwartz MA, Bokoch GM. Activation of Rac and Cdc42 by integrins mediates cell spreading. Mol Biol Cell. 1998;9(7):1863–71. doi: 10.1091/mbc.9.7.1863 9658176 PMC25428

[51] Hakeda-Suzuki S, Ng J, Tzu J, Dietzl G, Sun Y, Harms M, et al. Rac function and regulation during Drosophila development. Nature. 2002;416(6879):438–42. doi: 10.1038/416438a 11919634

[52] Bieling P, Rottner K. From WRC to Arp2/3: collective molecular mechanisms of branched actin network assembly. Curr Opin Cell Biol. 2023;80:102156. doi: 10.1016/j.ceb.2023.102156 36868090

[53] Rottner K, Stradal TEB, Chen B. WAVE regulatory complex. Curr Biol. 2021;31(10):R512–7. doi: 10.1016/j.cub.2021.01.086 34033782 PMC8882368

[54] Shekhar S, Kerleau M, Kühn S, Pernier J, Romet-Lemonne G, Jégou A, et al. Formin and capping protein together embrace the actin filament in a ménage à trois. Nat Commun. 2015;6:8730. doi: 10.1038/ncomms9730 26564775 PMC4660058

[55] Shekhar S, Pernier J, Carlier M-F. Regulators of actin filament barbed ends at a glance. J Cell Sci. 2016;129(6):1085–91. doi: 10.1242/jcs.179994 26940918

[56] Barbieri JT, Riese MJ, Aktories K. Bacterial toxins that modify the actin cytoskeleton. Annu Rev Cell Dev Biol. 2002;18:315–44. doi: 10.1146/annurev.cellbio.18.012502.134748 12142277

[57] Colinet D, Schmitz A, Depoix D, Crochard D, Poirié M. Convergent use of RhoGAP toxins by eukaryotic parasites and bacterial pathogens. PLoS Pathog. 2007;3(12):e203. doi: 10.1371/journal.ppat.0030203 18166080 PMC2156102

[58] Lemichez E, Aktories K. Hijacking of Rho GTPases during bacterial infection. Exp Cell Res. 2013;319(15):2329–36. doi: 10.1016/j.yexcr.2013.04.021 23648569

[59] Stradal TEB, Schelhaas M. Actin dynamics in host-pathogen interaction. FEBS Lett. 2018;592(22):3658–69. doi: 10.1002/1873-3468.13173 29935019 PMC6282728

[60] Boyer L, Paquette N, Silverman N, Stuart LM. Bacterial effectors: learning on the fly. Adv Exp Med Biol. 2012;710:29–36. doi: 10.1007/978-1-4419-5638-5_4 22127883 PMC3427734

[61] Labrosse C, Eslin P, Doury G, Drezen JM, Poirié M. Haemocyte changes in D. Melanogaster in response to long gland components of the parasitoid wasp Leptopilina boulardi: a Rho-GAP protein as an important factor. J Insect Physiol. 2005;51(2):161–70. doi: 10.1016/j.jinsphys.2004.10.004 15749101

[62] Moreau SJM, Asgari S. Venom proteins from parasitoid wasps and their biological functions. Toxins (Basel). 2015;7(7):2385–412. doi: 10.3390/toxins7072385 26131769 PMC4516919

[63] Trainor JE, Kr P, Mortimer NT. Immune cell production is targeted by parasitoid wasp virulence in a drosophila-parasitoid wasp interaction. Pathogens. 2021;10(1):49. doi: 10.3390/pathogens10010049 33429864 PMC7826891

[64] Rizki RM, Rizki TM. Effects of lamellolysin from a parasitoid wasp on Drosophila blood cells in vitro. J Exp Zool. 1991;257(2):236–44. doi: 10.1002/jez.1402570214 1899269

[65] Rizki TM, Rizki RM. Lamellocyte differentiation in Drosophila larvae parasitized by Leptopilina. Dev Comp Immunol. 1992;16(2–3):103–10. doi: 10.1016/0145-305x(92)90011-z 1499832

[66] Lam VK, Tokusumi T, Cerabona D, Schulz RA. Specific cell ablation in Drosophila using the toxic viral protein M2(H37A). Fly (Austin). 2010;4(4):338–43. doi: 10.4161/fly.4.4.13114 20798602 PMC3174484

[67] Rötte M, Höhne MY, Klug D, Ramlow K, Zedler C, Lehne F, et al. CYRI controls epidermal wound closure and cohesion of invasive border cell cluster in Drosophila. J Cell Biol. 2024;223(12):e202310153. doi: 10.1083/jcb.202310153 39453414 PMC11519390

[68] Yanagawa S, Lee JS, Ishimoto A. Identification and characterization of a novel line of Drosophila Schneider S2 cells that respond to wingless signaling. J Biol Chem. 1998;273:32353–9. 9822716 10.1074/jbc.273.48.32353

[69] Nagel BM, Bechtold M, Rodriguez LG, Bogdan S. Drosophila WASH is required for integrin-mediated cell adhesion, cell motility and lysosomal neutralization. J Cell Sci. 2017;130(2):344–59. doi: 10.1242/jcs.193086 27884932

[70] Schillers H, Rianna C, Schäpe J, Luque T, Doschke H, Wälte M, et al. Standardized nanomechanical atomic force microscopy procedure (SNAP) for measuring soft and biological samples. Sci Rep. 2017;7(1):5117. doi: 10.1038/s41598-017-05383-0 28698636 PMC5505948

[71] Carl P, Schillers H. Elasticity measurement of living cells with an atomic force microscope: data acquisition and processing. Pflugers Arch. 2008;457(2):551–9. doi: 10.1007/s00424-008-0524-3 18481081

[72] Schillers H, Medalsy I, Hu S, Slade AL, Shaw JE. PeakForce Tapping resolves individual microvilli on living cells. J Mol Recognit. 2016;29(2):95–101. doi: 10.1002/jmr.2510 26414320 PMC5054848

