# Supplementary figures and images for: Cdc42 and Rac2 act through the formin-like Frl/FMNL to control lamellocyte shape and encapsulation of parasitoid wasp eggs in Drosophila
Source: PLoS Pathog. 2026 Jul 20;22(7):e1014440. doi: 10.1371/journal.ppat.1014440 (PMC13395357; doi:10.1371/journal.ppat.1014440)

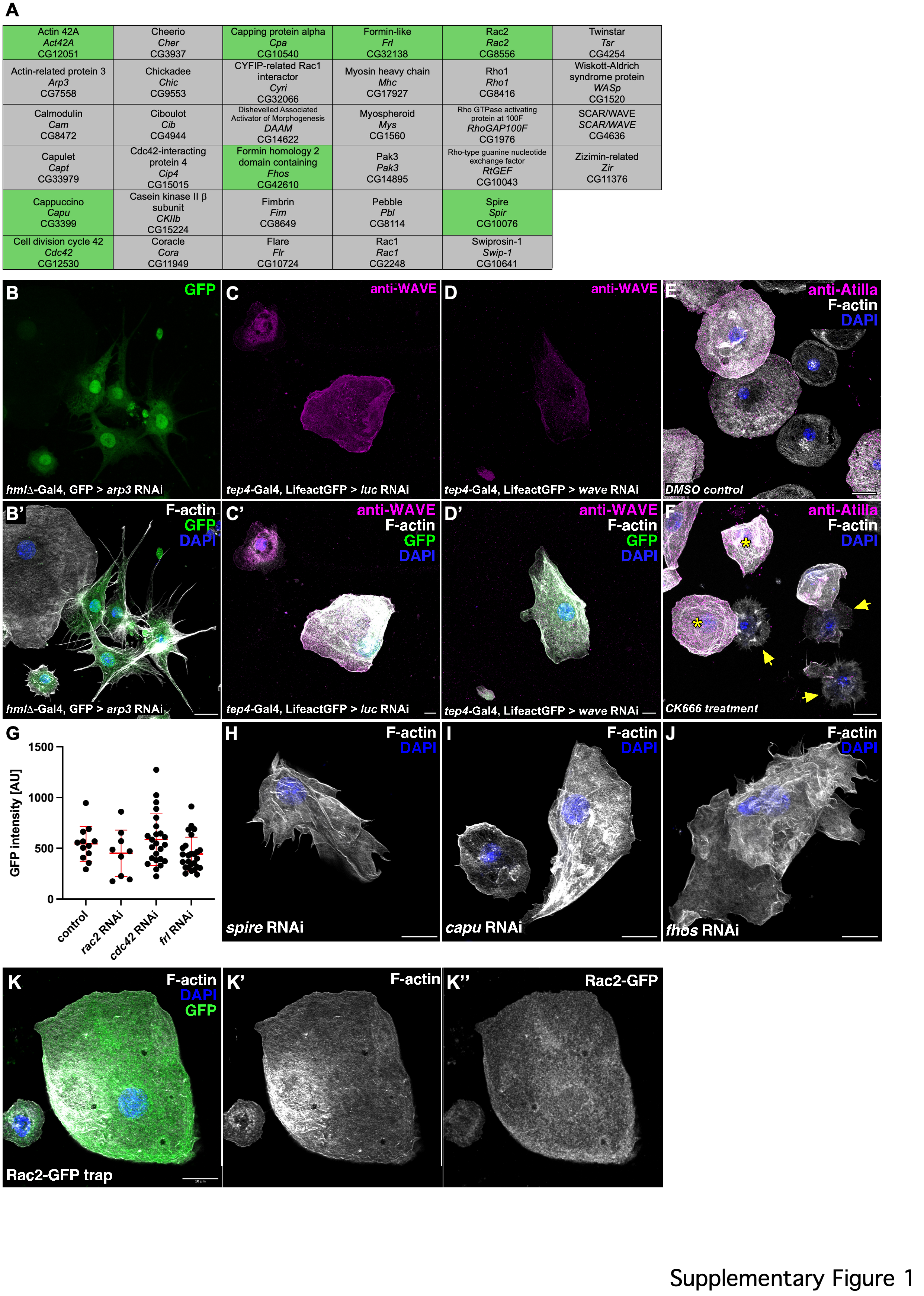

Supplement: S1 Fig — Genes, whose knockdown resulted in obvious changes in lamellocyte morphology are marked in green. Genes, where knockdown was tested but did not result in obvious morphology changes are marked in grey. For every gene, a representative number of cells from two independent experiments were screened for atypical morphology. (B-B’) The same RNAi construct that did not show a morphological phenotype in lamellocytes (see Fig 3E) causes long cytoskeletal protrusions in plasmatocytes under the control of hmlΔ-Gal4. Expression of the Gal4 driver is marked by GFP. Lamellocytes are rare but can occur in uninfected animals and are negative for hmlΔ-Gal4. F-actin is stained with phalloidin (grey) and nuclei with DAPI (blue). Scale bar: 10 µm. Representative images from 3 independent biological replicates, 10 animals each. (C-D’) Immunofluorescence staining showing efficient knockdown of WAVE in lamellocytes. (C-C’) In the control condition, lamellocytes strongly express WAVE as shown by specific antibody staining (magenta). (D-D’) This staining is largely lost upon knockdown of WAVE. F-actin is stained with phalloidin (grey) and nuclei with DAPI (blue). Scale bars: 10 µm. Representative images from 3 independent biological replicates, 10 animals each. (E-F) Lamellocyte morphology is not affected by treatment with the Arp2/3 inhibitor CK-666. (E) Lamellocytes (marked by antibody staining against Atilla (magenta)) and plasmatocytes from hopTum-l larvae after treatment with DMSO (control). (F) Lamellocytes and plasmatocytes from hopTum-l larvae after treatment with CK-666 (200µmol/l). Note the long cytoskeletal extensions in plasmatocytes (arrows) while lamellocytes are unaffected (asterisks). F-actin is stained with phalloidin (grey) and nuclei with DAPI (blue). Scale bars: 10 µm. Representative images from 3 independent biological replicates, 10 animals each. (G) Quantification showing that expression level of the tep4-Gal4 driver (measured by the intensity of Lifeact-GFP) is unch [file ppat.1014440.s001.tif]

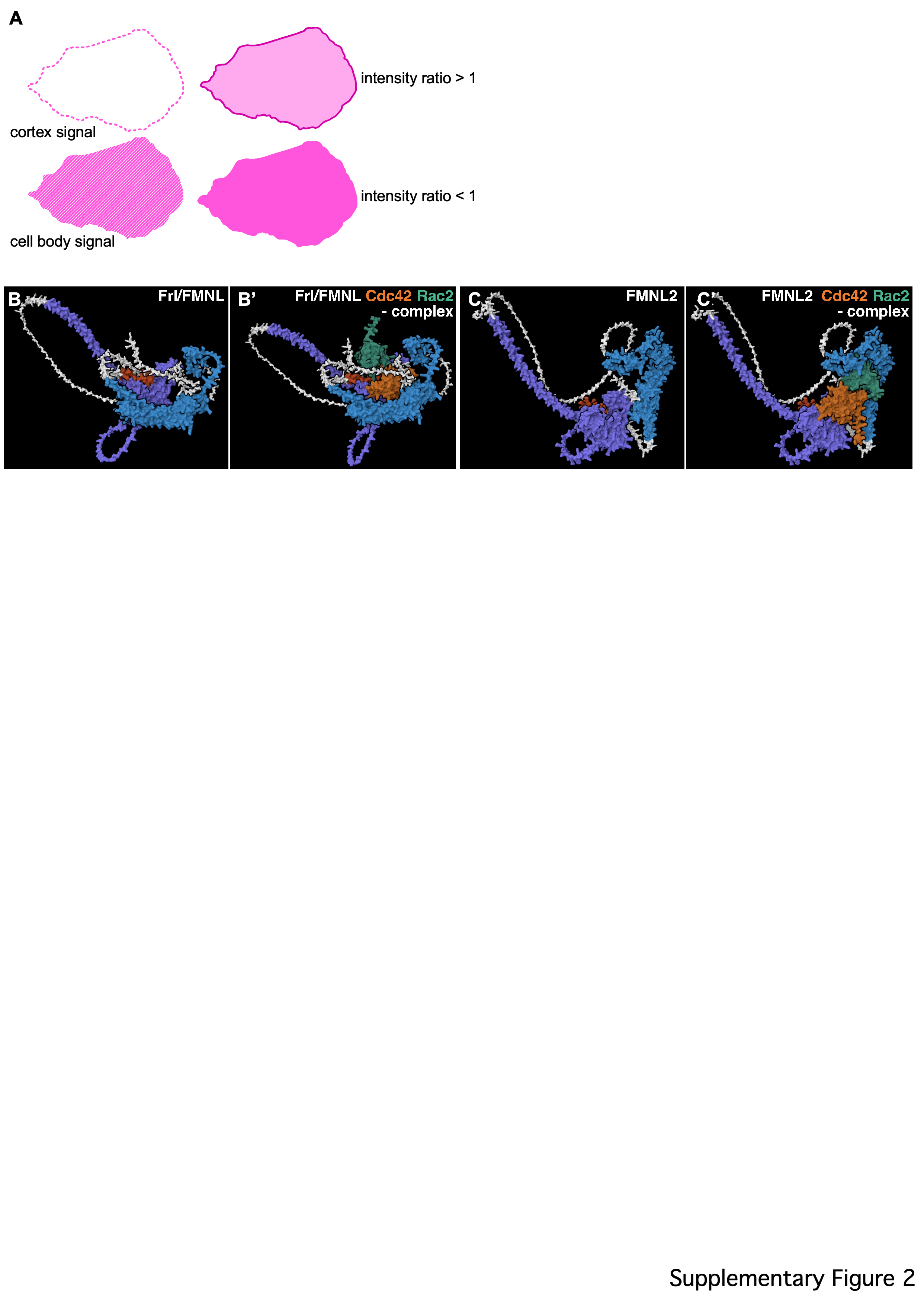

Supplement: S2 Fig — (B) In silico AlphaFold 3 prediction of Drosophila Frl/FMNL structure. The diaphanous autoregulatory domain (DAD) is marked in red, FH2 domain is marked in blue and GTPase binding domain is marked in purple. (B’) In silico AlphaFold 3 interaction prediction of Drosophila Frl/FMNL (same color code as in G), Cdc42 (orange) and Rac2 (green). Predicted template modelling score (pTM) = 0.42. (C) In silico AlphaFold 3 structural prediction of human FMNL2 (same color code as for the Drosophila homolog in G). (C’) In silico AlphaFold 3 interaction prediction between human FMNL2 (same color code as for the Drosophila homolog in G), Cdc42 (orange) and Rac2 (green). pTM = 0.4. (TIF) [file ppat.1014440.s002.tif]

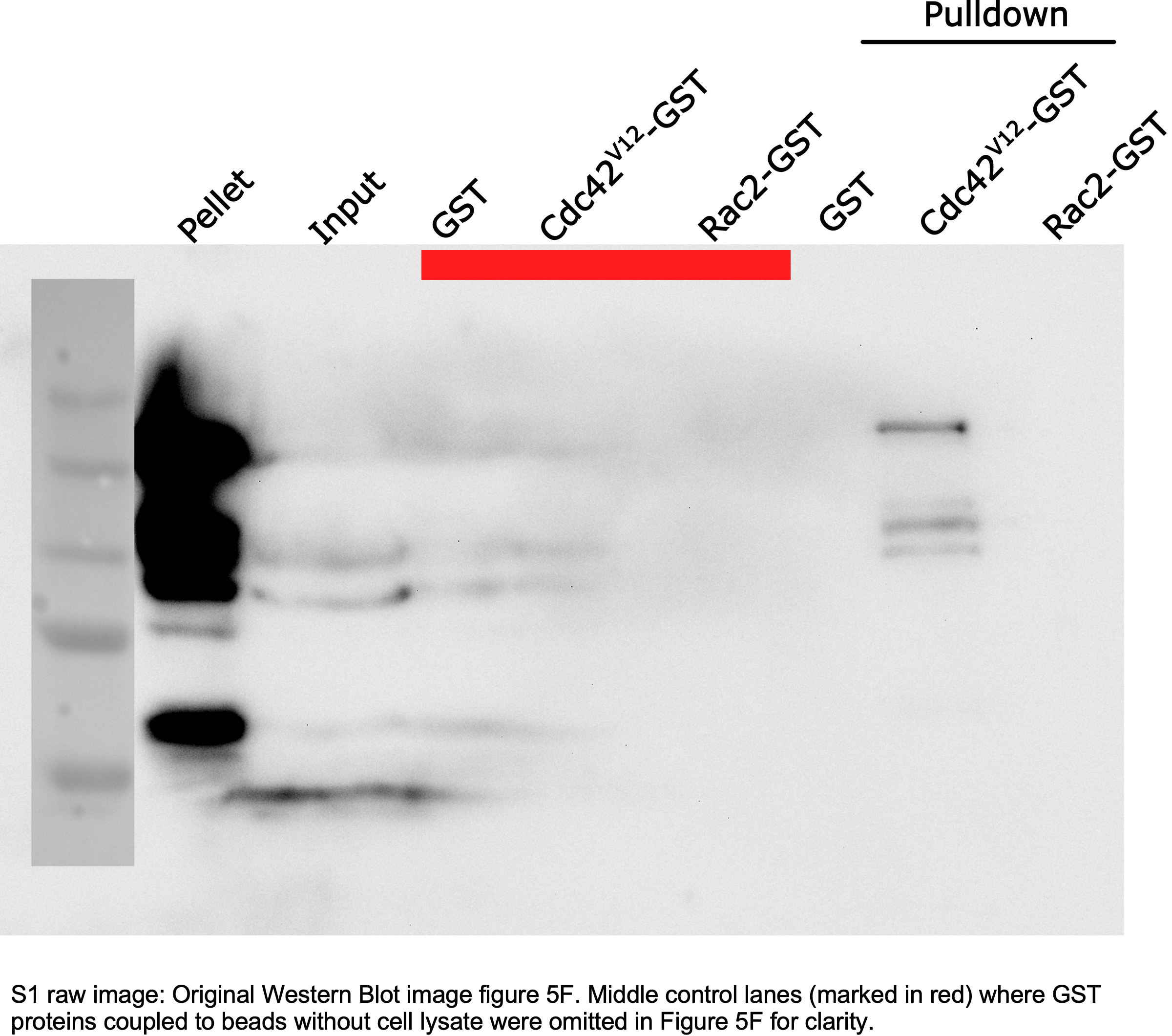

Supplement: S1 Raw Gel — The middle control lanes (marked in red), in which GST proteins were coupled to beads without cell lysate, were omitted from Fig 5F for clarity. (TIF) [file ppat.1014440.s003.tif]
