# Supplementary material for: Cdc42 and Rac2 act through the formin-like Frl/FMNL to control lamellocyte shape and encapsulation of parasitoid wasp eggs in Drosophila
Source: PLoS Pathog. 2026 Jul 20;22(7):e1014440. doi: 10.1371/journal.ppat.1014440 (PMC13395357; doi:10.1371/journal.ppat.1014440)
Supplement: Attachment — Submitted filename: Response to reviewers 11.05.26..docx [file ppat.1014440.s015.docx]

We thank all reviewers for their careful and very constructive comments.

Let me briefly highlight the **most important points** that we have addressed before I provide a detailed point-to-point- response to the referees’ comments.

1) **First**, we now provided all **experimental details in the Methods section and figure legends** including the number of technical and biological replicates. We also added a paragraph on statistical analysis to the Methods section.

2) **Second**, we further **provided evidence that WAVE-Arp2/3 function has no important role in the morphology of lamellocytes**. In detail, we showed that the same RNAi transgenes used in lamellocytes dramatically affect the cell morphology of plasmatocytes using a **plasmatocyte-specific Gal4 driver** (*hmlΔ*-Gal4). We also performed **immunostaining for endogenous WAVE** which is dramatically reduced in lamellocytes depleted for WAVE. In addition, **we inhibited Arp2/3 using the allosteric inhibitor CK666**, which only affects plasmatocyte, but not lamellocyte morphology. These new experimental (control) data are included in a new figure (Supplementary figure S1)

3) **Third,** we **improved** **readability** **of the manuscript** and presentation of the results **and methods.**

**4) Forth,** we performed **atomic force microscopy (AFM) analysis** and show for the first time that lamellocytes are more rigid than smaller, migrating plasmatocytes. More importantly, we further show that **lamellocytes become significantly softer during spreading** when the cortical actin cytoskeleton reorganizes and drives lamellipodia-like protrusions. These new exciting data are included in a new figure 1.

5) **Fifth**, we further **improved our live-imaging analysis** on lamellocyte spreading on wasp eggs/larvae. We are now able to better visualize the spreading behavior and dynamic F-actin driven membrane protrusions of lamellocytes in more detail. These new data are also included in the new figure 1 as well as two new supplementary movies (M5 and M6).

As you will see, we have also addressed **all other reviewer’s comments** and provide a set of new experiments (including GST pull downs) and controls including one new main figure, three changed main figures, one new supplementary figure and three new supplementary movies.

Below I provide a detailed response to the referees’ comments.

**Comments:** 

**Reviewers' Comments:**

Reviewer's Responses to Questions

**Part I - Summary**

Please use this section to discuss strengths/weaknesses of study, novelty/significance, general execution and scholarship.

**Reviewer #1:** The manuscript by Molitor and Bogdan investigates the cell morphology of lamellocytes, an interesting class of derived immune cells induced during the Drosophila anti-parasitoid response. The reporter research analyzed the roles of the cytoskeleton and its regulators in determining lamellocyte morphology and function. Molitor and Bogdan arrived at the interesting finding that lamellocyte morphology determination is unique when compared to other classes of Drosophila immune cells, and make important strides towards identifying and characterizing this mechanism. This paper is significance in the study of host-parasite interactions and immune cell biology: their work provides a close characterization of an interesting immune cell type, which to date has largely been studied in the contexts of cell type specification and immune function. Their characterization of lamellocyte cell morphology study adds an interesting new dimension to the field.

We thank the reviewer for the positive feedback.

**Reviewer #2:** This is an interesting study that investigates the cytoskeleton and its regulators in specialized Drosophila immune cells known as lamellocytes. The authors conduct an RNAi screen which results in the implication of a number of molecules in normal morphology of these cells. Functional validation is carried out for rac2 and fril through investigation of parasitoid wasp egg encapsulation by mutants. A weakness of the study is that the significance of negative results for the WAVE Arp2/3 are overstated. These experiments are conducted with RNAi, and the authors do not validate that the targeted proteins are indeed depleted by the RNAi methodology. 1) To substantiate these claims, mutant alleles would need to be investigated, perhaps with MARCM clones if viability of larvae is an issue) or validation of depletion is necessary. If suitably revised the study would be of interest to the readers of PLOS Pathogens.

We thank the reviewer for these constructive comments. We further provided evidence that WAVE-Arp2/3 function has no important role in lamellocyte morphology. In detail, we showed that the same RNAi transgenes used in lamellocytes dramatically affect the cell morphology of plasmatocytes using a plasmatocyte-specific Gal4 driver (*hmlD*-Gal4). We also performed immunostaining for endogenous WAVE which is dramatically reduced in lamellocytes depleted for WAVE. In addition, we inhibited Arp2/3 using the allosteric inhibitor CK666, which only affects plasmatocyte, but not lamellocyte morphology. These new experimental (control) data are included in a new figure (Supplementary figure S1)

**Reviewer #3:** Cdc42 and Rac2 acts through the formin-like Frl/FMNL to control lamellocyte shape and encapsulation of parasitoid wasp eggs in Drosophila

In this manuscript the authors aim to understand which proteins functionally associated with the microfilament cytoskeleton are important in controlling the shape of lamellocytes and whether these proteins are needed for lamellocytes to wrap around the wasp egg. After describing the actin filament (and tubulin) networks in lamellocytes and their shapes and protrusions from wasp-infested hosts, the authors used RNAi lines for candidate genes and cell-specific markers to identify a putative protein complex of Frl/FMNL, Rac2, and Cdc42. They show that Frl/FMNL is localized at cortex and its localization depends on Cdc42 and Rac2 functions. All three proteins are needed for lamellocyte shape and knockdown of Frl/FMNL and Rac2 caused lamellocyte wasp encapsulation defects. These conclusions are largely supported by the data they provide. The writing, figures, tables and movies are clear, showing varied hemocyte morphologies. 2) Quantification of the results are not explained enough to know how reproducible the results are. The novel aspects of the study could be highlighted in the context of a hypothesis.

We apologize for the unclear description of quantifications and the lack of experimental details. We now provided all experimental details in the Methods section and figure legends including the number of technical and biological replicates. We also added a paragraph on statistical analysis to the Methods section.

**Part II – Major Issues: Key Experiments Required for Acceptance**

Please use this section to detail the key new experiments or modifications of existing experiments that should be absolutely required to validate study conclusions.

Generally, there should be no more than 3 such required experiments or major modifications for a "Major Revision" recommendation. If more than 3 experiments are necessary to validate the study conclusions, then you are encouraged to recommend "Reject".

**Reviewer #1:** My only major issue is with the notable lack of experimental detail in the Methods section and figure legends. The figure legends present an N value and statistical significance for many quantitative experiments, but:

1. N is never defined. Is N the number of cells imaged, larvae dissected, or biological replicates? If N is the number of cells, how many cells were imaged per larvae per replicate? It’s essential for the authors to clarify how many independent samples were analyzed.

As mentioned above, we added further experimental details of quantifications to the respective figure legends, including the number of technical and biological replicates and what they represent in the context of the experiment. We also added a paragraph on statistical analysis to the Methods section.

2. Likewise, p values are provided **but the statistical tests** used to determine these values are never specified.

We added the statistical tests to the figure legends and added a paragraph on statistical analysis to the Methods section.

Additionally, data are presented for measures of cell circularity and the cortex:cell body ratio of Frl staining, but the underlying methodology is not described anywhere in the manuscript. Again, the authors must provide essential details that would allow for experimental replication.

We added a paragraph on '*image analysis*' to the Methods section where we describe our measurements of cell circularity, area, fluorescence intensities and the Frl cortex:cell body ratio.

All of these issues with methods reporting make it difficult to judge the strengths and limitations of the presented data, and to assess the degree that data support the conclusions of the manuscript.

We also further improved the readability of the manuscript and presentation of the results and methods.

**Reviewer #2:** As already noted claims that WAVE-Arp 2/3 complex are not required are weakly supported by the evidence. Negative results with RNAi must be interpreted with caution. Further evidence is necessary to support these claims. Alternatively, the authors should discuss the caveats of the methodology and state that a role for this complex cannot be ruled out using RNAi.

We thank the reviewer for these constructive comments. We indeed provided further evidence that WAVE-Arp2/3 function has no important role in lamellocyte morphology. In detail, we showed that the same RNAi transgenes used in lamellocytes dramatically affect the cell morphology of plasmatocytes using a plasmatocyte-specific Gal4 driver (hmlΔ-Gal4). We performed immunostaining for endogenous WAVE which is dramatically reduced in lamellocytes depleted for WAVE. In addition, we inhibited Arp2/3 using the allosteric inhibitor CK666, which only affects plasmatocyte, but not in lamellocyte morphology. These new experimental (control) data are included in a new figure (Supplementary figure S1)

Further, we changed the wording in the text to not overstate the role of WAVE and Arp2/3 in this context.

**Reviewer #3:** In the following review, feedback is provided for the reviewers to consider:

The authors screen for the functional requirement of multiple proteins, predicted or known to be expressed at high levels in lamellocyte shape and function. This is reasonable starting point. To make this story more broadly interesting, the authors could articulate or define precise question(s) and consider formulating a hypothesis. For example, based on the interpretation of the data (Discussion lines 306-307), the picture that emerges is that lamellocytes are stiff, they do not appear to form lamellopodia, and their movement in vivo may be due to passive flow. Are these results novel or were they expected/unexpected? And how good is the evidence in support of this interpretation?

We thank the reviewer for this comment. We now performed atomic force microscopy (AFM) analysis and showed for the first time that lamellocytes are more rigid than smaller, migrating plasmatocytes. More importantly, we further show that lamellocytes become significantly softer during spreading when the cortical actin cytoskeleton reorganizes and drives lamellipodia-like protrusions. These new exciting data are included in a new figure 1. Accordingly, we changed the discussion to better represent the novelty and relevance of these findings.

(1) There is significant variation in lamellocyte morphologies. Is the expression pattern of Tep4 GAL4 uniform in all lamellocyte types? If this has been studied, please mention it with reference.

We agree with the reviewer that lamellocyte size and morphology can very. We are using the Tep4-Gal4 to visualize lamellocytes for several years (see also our paper Hirschhäuser et al., 2023, Development). Tep4-Gal4 drives expression uniformly in all types of lamellocytes.

(2) Does the binding of lamellocytes to Concanavalin A bias the cell population being observed?

Concanavalin A (ConA) is a lectin that binds to cell surface glycoproteins of all cells, even *Drosophila* Schneider 2 (S2) cells which are primarily non-adhesive and grow in suspension different from adhesive S2R+ cells.

(3) Can we say with some confidence that RNAi-mediated knockdown of these important proteins does not alter (a) hemocyte identities, and (b) expression levels of GAL4 driver(s)? Please comment on this and provide evidence.

We thank the reviewer for this comment. None of these RNAi-mediated knockdowns significantly alter hemocyte identities or the expression level of the Tep4-Gal4 driver in lamellocytes. We included this important information in the main text (line 242-244 and in the new Supplementary figure S1G.

(4) Line 134-136: … isolated lamellocytes have a very rigid disc-shaped morphology with no prominent protrusion dynamics when plated on glass surfaces. What does “very rigid” mean? If rigidity was not measured, then the authors should rephrase their interpretation to qualify what they mean and how it can be tested.

As mentioned above, we now performed atomic force microscopy (AFM) analysis and showed for the first time that lamellocytes are more rigid than smaller, migrating plasmatocytes. More importantly, we further show that lamellocytes become significantly softer during spreading when the cortical actin cytoskeleton reorganizes and drives lamellipodia-like protrusions. These new exciting data are included in a new figure 1. Accordingly, we changed the discussion to better represent the novelty and relevance of these findings.

(5) Explain more clearly is the significance of the results in Fig 1 (for example tubulin results) to the rest of the paper. Do the identities of the cells change upon the introduction of these drugs/toxins?

We rephrased the results section to better integrate the results in new Figure 2 to the rest of the manuscript. We never observed a change of cell identities upon drug treatments. Overall, it is very unlikely that exposure to these drugs for 1 hour before fixation would be sufficient time to induce cell fate changes. To clarify this point, we rephrased the relevant paragraph in the Methods section.

(6) Movie S3: What are the bright signals internal to the cells? Are they ruffles or some intracellular organelles? Is it possible for the authors to interpret the internal morphology a little more?

We thank the reviewer for this comment. Yes, indeed these are very interesting membrane structures (ruffles) observed not only in living cells by spinning disk microscopy but also in fixed cells using atomic force microscopy (see new Figure 1C)

We rephrased the results to better describe this movie. Further, a newly added 3D reconstruction shows that these bright signals are indeed actin-rich membrane ruffles that protrude from the cell surface (new supplementary movie M4).

(7) What is Cpa’s role in encapsulation?

We thank the reviewer for this comment. We indeed looked at cpa mutants and found that that cpa is also required for cellular encapsulation, quantified in figure 5D, E. Interestingly, previous studies demonstrated that formins such as FMNL2 and capping protein functionally coregulate filament barbed-end assembly to drive protrusion dynamics (Shekhar et al., 2015; Shekhar et al., 2016). We also discussed this in the discussion.

Fig 3: Make a schematic showing cortex and body and the cortex-body ratio for a couple of different cell shapes, shown in the Figure panel, to illustrate the Result. In some cases, lamellocyte shapes are so atypical that this point needs to be clarified a bit more.

We agree with the reviewer and included an illustration showing how we quantified signals at the cortex, body and the cortex-body ratio, now included in supplementary figure S2:

-In Fig 3A, B: the confocal microscopy image of a lamellocyte expressing the Rac/Cdc42-sensor MBT-GFP shows somewhat higher signal around the nucleus than closer to the membrane, almost complementary to the anti-Frl straining. Is this true, and if so, is it observed consistently or just in the examples provided here?

Yes, indeed MBT-GFP co-localizes with F-actin around the nucleus.

Fig 4: Methods for the protein models and interaction predictions: Clarify what is newly done in this study. Add information that tells us how reliable these interaction predictions. Is it possible to predict if and how the virulence factor in L. boulardi venom might affect these interactions?

We now included the pTM values of predicted trimeric Fr/FMNL-Cdc42-Rac2 complexes in the figure legend, which assess the accuracy of the overall structure of the protein complex. With a value of 0.5, the pTM value for RhoGAP-Rac2 is slightly higher than that of predicted Frl/FMNL-Cdc42-Rac2complexes, which are 0.42 and 0.42, respectively. For small proteins like RhoGTPases, the pTM scores are usually lower, even if the structure is correct. We therefore decided to include these protein models and interaction predictions in a new supplementary figure S2.

Statistics: Add a section in the Methods describing how cells were selected (randomly or not). What was the minimum number of cells used in the analyses, which tests were done and how? How many technical and biological replicates were done for each quantification? For each result where quantification is provided, add relevant details in the Fig legend for the reader to assess the strength of the result. Biological replicates must be included for quantification and subsequent publication.

We added experimental details of quantifications to the respective figure legends, including the number of technical and biological replicates and what they represent in the context of the experiment. We further added a paragraph on statistical analysis to the Methods section that also describes the selection of cells for quantification.

**Part III – Minor Issues: Editorial and Data Presentation Modifications**

Please use this section for editorial suggestions as well as relatively minor modifications of existing data that would enhance clarity.

**Reviewer #1:** For the lamellocyte: wasp egg interaction studies (in lines 143-147), how were the wasp eggs collected? It’s been previously established (e.g., Russo et al., Parasitology, 1996; Mortimer et al., Plos Pathogens, 2012) that plasmatocytes bind directly to the wasp egg surface, and that the lamellocytes are more likely to interact with the plasmatocyte layer in capsule formation.

Depending on the egg collection, this plasmatocyte layer may not be present, e.g. if eggs are isolated directly from the ovaries. This method should be more clearly specified, and the conclusions considered carefully if the cell layer is absent. Also, lines 309-310 refer to lamellocyte-egg contacts and may need to be amended.

We apologize for any confusion. We now better explained the method in the Methods section. Briefly, wasp eggs were dissected from L2 larvae 24 hours after wasp infestation in dissection medium. We further improved our live-imaging analysis on lamellocyte spreading on wasp eggs/larvae. We are now able to better visualize the spreading behaviors and dynamic F-actin driven membrane protrusions of lamellocytes in more detail. We also imaged lamellocyte directly spreading on parasitoid wasp eggs isolated from infested L2 fly larvae expressing a dual reporter msn-Cherry/eaterGFP reporter (Anderl et al., 2016). We found both lineages, lamellocytes (mCherry positive), plasmatocytes (GFP) as well as intermediates expressing both markers attached to the eggs (Figure 1F, F'; supplementary movie M5). Once attached to a developing parasitic wasp larva, mCherry-positive spread on the wasp eggs and underwent a similar change in cell morphology like isolated lamellocytes plated on Concavalin A (included new Figure 1F, F'; new supplementary movie M5). Some intermediates were dividing while attached to a wasp egg as previously reported (Anderl et al., 2016). We found no evidence for distinct plasmatocyte layer in capsule formation which is used for lamellocyte attachment to the egg as previously published. Again, both cell types were found simultaneously on the eggs.

The new imaging data are included in the new figure 1 as well as two new supplementary movies (M5 and M6).

Additionally, the descriptions of Suppl. movies M4+M5 are confusing (lines 783-790). It’s unclear how the conclusions are reached. These interactions seem very short lived, in contrast to capsule formation. In M4 the parasitoid egg also seems to be considerably bigger than the purported wasp larva in M5 even though the images taken at the same scale.

As mentioned above, we greatly improved our live-imaging analysis on lamellocyte spreading on wasp eggs/larvae. We are now able to better visualize the spreading behaviors and dynamic F-actin driven membrane protrusions of lamellocytes in more detail. The new imaging data are included in the new figure 1 as well as two new supplementary movies (M5 and M6).

It would be helpful to provide more context for some of the reported screen results. For instance, lines 211-212 list several genes with phenotypes. Are these genes known interactors of Frl? Is this a new connection between these genes?

As suggested by reviewer #3 we further looked at *cpa* mutants and found that that cpa is also required for cellular encapsulation, quantified in new figure 5D, E. Interestingly, previous studies demonstrated that formins such as FMNL2 and capping protein functionally coregulate filament barbed-end assembly to drive protrusion dynamics (Shekhar et al., 2015; Shekhar et al., 2016). We also discussed this in the discussion section.

Similarly, lines 213-214 describe two Diaphanous-related formin genes with no morphological phenotypes. The text previously described Frl as the sole member of the Diaphanous-related formin subfamily in Drosophila (lines 207-208). This relationship needs to be clarified.

We apologize for any confusion and changed this in the main text. The *Drosophila* genome encodes six formin proteins. While mammals possess three closely related Diaphanous-related formin genes (DIAPH1, DIAPH2, and DIAPH3), *Drosophila* only has a single dia gene.

In line 354, I think that saying that use of structure simulations “confirmed” binding interactions is misleading. Overall, I’m not convinced that the structure simulations add anything to the manuscript, and could be removed, but at a minimum they should be better described.

As mentioned above, we performed GST pull down experiments and confirmed a significant interaction between Cdc42 and Frl/FMNL, which were expressed with a GFP tag in S2R+ cell culture (included in new Figure 5F). However, the majority of this GFP-tagged Frl/FMNL protein were found in the insoluble lysate fraction despite increasing detergent concentration up to 2%. AlphaFold 3 prediction indeed confirmed that Frl/FMNL is able to directly bind Cdc42 but not Rac2. AlphaFold 3 also predicts Cdc42/Rac2 heterodimers that nicely fit into the FH3 binding pocket, both for Drosophila Frl/FMNL and human FMNL2. Thus, such Cdc42/Rac2 heterodimers might act as a bridge, forming Frl/FMNL complexes with specialized cellular roles. We now included these predictions in the result section and discussed it in the discussion.

I also noted some issues with terminology.

First, the term avirulent is used, e.g., line 269. It is not accurate to say that this wasp strain is avirulent, given that the data show that most of the wasps are evading encapsulation and hatching in the host (lines 270-271). The use of avirulent would imply that the wasps have no immune evasive (or other) activity against the host.

The term avirulent is often used to describe the G486 strain in the literature to contrast it with other strains with near absolute immune response evasion. We agree that this is not an ideal use of the term and have replaced it with "*low-virulent*" in the text.

Similarly, I don’t think it’s correct to characterize the eggs as properly encapsulated in line 274. The described interactions would not be sufficient to conclude that encapsulation is occurring, again particularly given that most of the wasps are not being encapsulated. Proper encapsulation would specifically refer to a wasp egg completely surrounded by a melanized capsule and killed by this immune reaction. I completely agree with the authors that the differences in lamellocyte interaction with the wasp egg between genotypes are interesting and likely functionally relevant, but this alternative use of established terminology is misleading.

We agree with the reviewer. We showed that wasp eggs/larvae were only partially encapsulated by wild type lamellocytes. We changed the text accordingly.

Comments on the figures and legends:

As noted above, replication and statistical testing information need to be added to all figures where appropriate.

as mentioned above, we now provided all experimental details in the Methods section and figure legends including the number of technical and biological replicates. We also added a paragraph on statistical analysis to the Methods section.

Figure 1: Aspect is misspelled in the figure.

We apologize for this and changed this.

The use of “cell size” (lines 677,679) is potentially confusing. It would be better to stick with cell area as the data are presented.

We rephrased this to cell area.

Figure 2: A: What is meant by “% expressed”? It could be a normalized expression value, or the number of expressing cells per cluster, so please make the description more precise.

“% expressed” indicates the number of cells per cluster where the respective gene was detected. We clarified this point in the figure legends.

Also, please consider recoloring the average expression value, a single-color gradient is very difficult for many people with vision issues to interpret. Personally, I see the scale as grey for -1.0 and as a single red color for all values between -0.5 to 1.0.

We changed the color scale.

Figure 4: The reader’s ability to interpret panels B and C would be greatly aided by displaying all of channels individually as shown for A.

We changed this.

As noted above, I’m not sure that the structure models presented in F-G’ add to the story. At a minimum, the use of color in the model should be explained in the legend.

We apologize for this and we provided a better a description of colors to the figure legend.

Suppl. Figure 1: A: the grey boxed genes are not explained by the legend. I’m assuming these genes were tested but no phenotype was seen, but it’s not clear. It would also help assessment of the screen if number of replicates or individuals screened, and/or the criteria for phenotype determination were reported.

We apologize for this and we added more details to the figure legend.

Suppl. Movies: Several of the movies have different display titles within a viewer (I used QuickTime Player for Mac). It’s not a big deal, but for instance movie M6 is titled movie M3 in the viewer, so I wanted to pass this observation along in case it becomes confusing.

We renamed the movies accordingly.

**Reviewer #2: (No Response)**

**Reviewer #3**: Scale bars: Some panels are lacking scale bars, or maybe they are just not visible?

Typos:

-Line 135 has a typo (glass, not glas).

-Lines 138 and 152: The spelling of Concanavalin A is incorrect

-Line 150 and other places: The genotype Hoptum-l should be hopTum-l (of course with italics and superscript, as already used)

-Line 722: “ist” stained should be “is” stained

-Please check in all other places

We re-checked all scale bars and spellings. Generally, we include scale bars in the merge panels but not in individual channels.

While the use of gene symbol abbreviations ((Frl/FMNL) is fine, it feels a little tedious. The authors could consider picking one or the other, after having defined them in the Introduction. Consider rephrasing the Title to see if it works in the absence of the gene symbols. It might enhance the impact of the study.

We thank the reviewer for this suggestion. We decided to choose Frl/FMNL for most of the text after establishing it as the human homolog of *Drosophila* Frl in the introduction. Most previous and recent studies use gene symbol abbreviation Frl/FMNL, both in invertebrates and vertebrates.
