# Supplementary material for: Cdc42 and Rac2 act through the formin-like Frl/FMNL to control lamellocyte shape and encapsulation of parasitoid wasp eggs in Drosophila
Source: PLoS Pathog. 2026 Jul 20;22(7):e1014440. doi: 10.1371/journal.ppat.1014440 (PMC13395357; doi:10.1371/journal.ppat.1014440)
Supplement: Attachment — Submitted filename: Response to Reviewers 30.06.26.docx [file ppat.1014440.s016.docx]

| Philipps-Universität - 35037 Marburg  Robert L. Unckless, Ph.D. Academic Editor PLOS Pathogens |  | Institut für Physiologie und Pathophysiologie  Abteilung Molekulare Zellphysiologie  Prof. Dr. Sven Bogdan  Tel.: 06421 28-26816  Fax: 06421 28-68960  E-Mail: [bogdan@staff.uni-marburg.de](mailto:bogdan@staff.uni-marburg.de)  Sek.: Martina Stolte  Tel.: 06421 28-66495  E-Mail: stolte@staff.uni-marburg.de  Anschrift: Emil-Mannkopff-Str. 2 35037 Marburg  Web: http://www.uni-marburg.de/fb20/physiologie |
| --- | --- | --- |
|  | | Marburg, 30^th^ June 2026 |

**PPATHOGENS-D-25-03187R1 – manuscript revision**

Dear Dr. Unckless,

thank you very much again for giving us the opportunity to revise our manuscript entitled “Cdc42 and Rac2 acts through the formin-like Frl/FMNL to control lamellocyte shape and encapsulation of parasitoid wasp eggs in *Drosophila*” (PPATHOGENS-D-25-03187) and the reviewers for their careful and very constructive comments.

As you will see, we have addressed both major points of criticism: first, a more detailed description of the GST binding experiments and their limitations (R1), and second, a more precise explanation of the sample size and the biological replicates in our experiments (R3).

Below I provide a detailed response to the referees’ comments.

With best regards

Sven Bogdan

**Reviewers' Comments:**

Reviewer's Responses to Questions

Part I - Summary

Please use this section to discuss strengths/weaknesses of study, novelty/significance, general execution and scholarship.

**Reviewer #1:** This manuscript by Molitor et al., provides an interesting and well-supported characterization of the Drosophila lamellocyte, an interesting yet understudied immune cell type. The authors have done a commendable job of addressing the reviewers' concerns, and the additional experiments and clarifications have significantly improved their manuscript. I have one concern about the GST-binding experiments, a few minor comments for clarification.

We thank the reviewer #1 for the very positive response and we improved the description of the GST binding experiments and further described their limitations.

**Reviewer #3:** The authors have made considerable efforts to address reviewer comments and the revised manuscript is significantly stronger. Additional points are below for the authors to consider to strengthen the manuscript further.

We also thank the reviewer #2 for the very positive response and we further improved our description of the sample size and the biological replicates in our experiments.

**Part II – Major Issues: Key Experiments Required for Acceptance**

**Reviewer #1:** Lines 323-324. According to the Methods (lines 553-555), the GST constructs **for Cdc42 and Rac2 are not equivalent** (i.e., that the activating Cdc42[V12] mutation was used, alongside the wild type Rac2). This difference does not seem to be mentioned in the Results or Discussion, despite this change potentially influencing binding, particularly given the result that Cdc42[v12] binds to Frl/FMNL whereas Rac2[WT] doesn't.

We apologize for any confusion. According to our Methods, we indeed used an activatin Cdc42[V12]- and a wild type Rac2-GST fusion for technical reasons. We also generated an expression vector bearing an activating Rac2[V12] mutation but unfortunately, we were unable to purify any soluble protein. For this reason, we used wild type Rac2-GST fusion kindly provided by Jörg Grosshans (University of Marburg). We now better described the use Cdc42[V12]- and a wild type Rac2-GST fusion in the results and also discuss this difference potentially influencing Frl/FMNL binding in the Discussion.

**Reviewer #3:** 1. Quantification and replication: Maybe I missed this but I did not find how many biological replicates were performed for each experiment. Is a cell considered a technical replicate of another cell? Is a larva from the same vial a technical or biological replicate? The authors should clarify this point more rigorously as this information was requested by all reviewers. The manuscript should not be published without biological replicates for all experiments, each including multiple technical replicates. Statistical analysis should then be performed from data derived from these replicates.

We apologize for any confusion. We consider measurements from individual cells/wasp eggs as technical replicates (N) and separate (independent) crossings/vials as biological replicates. We specified this in more detail in all figure legends, explicitly mentioning the terms “technical/biological replicate” for clarity. Statistical analysis was always performed from data derived from these replicates.

Example of the kind of information that is missing: This is text from Fig. 1A legend: Elasticity of cells given as the Young`s modulus. Indentation experiments revealed that partially spread lamellocytes are stiffer than fully spread lamellocytes. N = 40 (plasmatocytes), 74 (partially spread lamellocytes) and 180 (fully spread lamellocytes) force-distance curves from three independent preparations; One-way ANOVA with Kruskal-Wallis test, ***: p < 0.001.

2. Is the number of cells presented derived from all three preparations? Were preparations made from one animal or multiple animals in the same vial or multiple animals from different vials? The authors need to be clear about how they define a replicate. Explain in one sentence how Young’s modulus informs us of cell elasticity. The values and variation in the Young’s modulus numbers need to be mentioned in the Results sections.

In this example, the given N-values are technical replicates (cells) pooled from all preparations (biological replicates). Preparations were made from multiple animals from one vial each. For each preparation, a separate vial was used. We rephrased our descriptions in the figure legends to clarify our definition of a replicate. We also added a more detailed description of Young’s modulus and its meaning in the context of cell elasticity and mentioned values and variation in the Young’s modulus numbers in the Figure legends.

Similar feedback needs to be applied to all experiments, whether they were quantified or not.

As mentioned above, we now applied this description for all experiments.

3. Starting from the Abstract (Remarkably, cell spreading does not depend on WAVE-Arp2/3-branched actin filament nucleation but rather requires the formin Frl (also known as FMNL) downstream of Rac2 and Cdc42 signaling.), the authors imply that these two mechanisms are alternative to each other for the same function (cell spreading in this case). But in the Discussion, they discuss this issue in the context of cell protrusion and cell migration. From a cell biology point-of-view aren’t these behaviors different and assay dependent? It would be helpful for the readers if the authors qualify these terms in the context of the assays and the biology/physiology of the cell.

We agree with the reviewer and have refined our abstract and our discussion to provide a more coherent cell biology point-of-view of our work.

4. Instead of saying “…. WAVE-Arp2/3 function has no important role in lamellocyte morphology, I would suggest rephrasing to say “ Arp2/3 has no apparent or detectable role/function in…”.

We rephrased this in the Discussion as follows "WRC-Arp2/3-branched actin nucleation has no apparent function in the formation of these thin, sheet-like cellular protrusions of lamellocytes (see line 415f).

5. The movies show that the number of lamellocytes around the wasp egg is very low; at most one lamellocyte is shown in representative movies. The authors need to acknowledge that these few lamellocytes (how many cells observed in movies from how many experiments?) may have partially escaped the effects of the venom.

We would like to draw reviewers’ attention to Supplementary Movies 5 and 6. Unlike Supplementary Movies 4 and 5 in our originally submitted manuscript, many more lamellocytes are attached to the wasp egg (Movie M5: 16 cells in the first and 18 in the last frame; Movie M6: 6 cells throughout the movie). This is representative of more than 5 experiments. We purposefully do not use fully encapsulated wasp eggs, as it is very hard to observe individual cell behavior in this case. We can not definitively say, how many of the observed lamellocytes are affected by wasp venom but we also do not discuss the effects of *L. boulardi* G486 venom on cell spreading.

6. For the pulldown experiments, describe the methods for protein expression and include references (or sources) for constructs (e.g., pUAST attB GFP-Frl). Separate this information from the steps used in the pull-down experiment itself.

We added more experimental details and references for constructs and the pulldown experiments.

For the Western blot Methods section, add more details to the antibodies used. Mention conditions for staining the Western blots.

We added details on antibodies and staining the Western blots.

In the Western blot Figure panel, show more clearly which antigens were tagged with GFP so it is easy to interpret the result. **Why are the two Western blots in separate pieces? Were the markers not run in the same gel as the three samples in the right-most lanes?**

The two images show different sections of the same gel. Some lanes were omitted for clarity. We uploaded the original unchanged western blot with lanes in between where GST proteins coupled to beads without cell lysate were loaded.

7. Line 501-502: For fixation, eggs were collected in a drop of medium on a silane prep slide. What medium? Give details of the “silane prep slide”

We further specified the medium and added more details on the silane prep slides in the Methods. Briefly, silanization of glass surfaces increases their hydrophobicity and this helps in staining wasp eggs directly on the slide.

8. The genotype Hoptum-l: According to Flybase, the symbol for the hopscotch gene is hop (h not capitalized). The Tum-l allele is dominant, hence the t in tum-l should be capitalized. Not the other way round.

We changed this to *hop^Tum-l^* throughout the manuscript.

**9. Movies 9 and 10 are dispensable.**

We agree with the reviewer and removed movies 9 and 10.

10. In general, the quality of the Discussion can be improved. Right now, various results are mentioned in isolation of each other, but not synthesized in a cohesive picture. For example, the issue of lamellocyte rigidity is not tied to the molecular data in the manuscript. Is it known for other cell types how actin modulators affect cell rigidity? The authors should try to include as many results in the discussion as possible. The discussion of lamellolysin in L. heterotoma is not relevant to this manuscript as it is not expected to be present in L. boulardi. The authors should consider removing it.

We agree with the reviewer and have refined our discussion to provide a coherent overview of our work. However, we still discuss briefly the earlier pioneering work on lamellolysin, a virulence factor from *Leptopilina heterotoma*, which specifically targets and destroys lamellocytes by disrupting their microtubule cytoskeleton (Rizki and Rizki, 1984; Rizki and Rizki, 1991; Rizki and Rizki, 1992). The exact nature of Lamellolysin has been never identified but these studies also highlight a key role of microtubule cytoskeleton in cellular immune defense as well as the remarkable molecular evolution and functional convergence of venom proteins in host-pathogen interactions. Thus, we feel that this adds to our experiments regarding the role of the microtubule cytoskeleton in lamellocytes.

Part III – Minor Issues: Editorial and Data Presentation Modifications

Please use this section for editorial suggestions as well as relatively minor modifications of existing data that would enhance clarity.

**Reviewer #1:** **The protocol for measuring circularity is still not clearly defined,** at least not for my understanding. I'm assuming it is linked to the cortical measurements, but the word "circularity" doesn’t appear in the methods section so I'm still not able to follow the approach. I think this just needs a more explicit description.

We refined the description of image analysis in the Methods section.

Line 338 includes the idea that “heterodimers might act as a bridge, forming Frl/FMNL complexes with specialized cellular roles.” The predicted ability to bind to heterodimers is intriguing, but I'm not sure what the authors' statement means by "act[ing] as a bridge".

We agree with the reviewer. We now better describe that Cdc42/Rac2 heterodimers may function as an additional regulatory mechanism rather "acting as a bridge" by differentially localizing the Frl/FMNL complexes or by differentially regulating intrinsic GTPase activity, as has previously been shown for Cdc42 and Rac1 homodimers (Zhang and Zheng, 1998).

Reviewer #3: Please see above.
