# Supplementary material for: Cdc42 and Rac2 act through the formin-like Frl/FMNL to control lamellocyte shape and encapsulation of parasitoid wasp eggs in Drosophila
Source: PLoS Pathog. 2026 Jul 20;22(7):e1014440. doi: 10.1371/journal.ppat.1014440 (PMC13395357; doi:10.1371/journal.ppat.1014440)
Supplement: Peer review history

# Peer review: Decision Letter 0

**DOI:** 10.1371/journal.ppat.1014440.r001

**Authors:** Jeffrey Dvorin, Robert Unckless

11 Mar 2026

-->PPATHOGENS-D-25-03187

Cdc42 and Rac2 acts through the formin-like Frl/FMNL to control lamellocyte shape and encapsulation of parasitoid wasp eggs in Drosophila

PLOS Pathogens

Dear Dr. Bogdan,

Thank you for submitting your manuscript to PLOS Pathogens. After careful consideration, we feel that it has merit but does not fully meet PLOS Pathogens's publication criteria as it currently stands. Therefore, we invite you to submit a revised version of the manuscript that addresses the points raised during the review process.

Please submit your revised manuscript by May 10 2026 11:59PM. If you will need more time than this to complete your revisions, please reply to this message or contact the journal office at plospathogens@plos.org. When you're ready to submit your revision, log on to https://www.editorialmanager.com/ppathogens/ and select the 'Submissions Needing Revision' folder to locate your manuscript file.

Please include the following items when submitting your revised manuscript:

* A letter that responds to each point raised by the editor and reviewer(s). You should upload this letter as a separate file labeled 'Response to Reviewers'. This file does not need to include responses to any formatting updates and technical items listed in the 'Journal Requirements' section below.

* A marked-up copy of your manuscript that highlights changes made to the original version. You should upload this as a separate file labeled 'Revised Manuscript with Track Changes'.

* An unmarked version of your revised paper without tracked changes. You should upload this as a separate file labeled 'Manuscript'.

If you would like to make changes to your financial disclosure, competing interests statement, or data availability statement, please make these updates within the submission form at the time of resubmission. Guidelines for resubmitting your figure files are available below the reviewer comments at the end of this letter.

We look forward to receiving your revised manuscript.

Kind regards,

Robert L. Unckless, Ph.D.

Academic Editor

PLOS Pathogens

Jeffrey Dvorin

Section Editor

PLOS Pathogens

Sumita Bhaduri-McIntosh

Editor-in-Chief

PLOS Pathogens

orcid.org/0000-0003-2946-9497

Michael Malim

Editor-in-Chief

PLOS Pathogens

orcid.org/0000-0002-7699-2064

**Additional Editor Comments :**

Thank you for submitting your manuscript entitled "Cdc42 and Rac2 acts through the formin-like Frl/FMNL to control lamellocyte shape and encapsulation of parasitoid wasp eggs in Drosophila" for consideration by PLOS Pathogens. We apologize for the delays in review. All three were enthusiastic about the work and feel that it would be a worthwhile contribution to the field. However, all three had important comments that should be addressed before publication. Most importantly, Reviewer #1 points out the general lack of detail in methods and figures which made assessing the work difficult. I encourage you to carefully revise the manuscript taking into account the various points made by reviewer #1. Reviewer #2 suggests that the WAVE Arp2/3 result might be overstated especially given the lack of validation of the RNAi results. Finally, Reviewer #3 suggests framing with a question for a broader audience, and several methodological and presentation changes to improve readability. We look forward to your revision.

**Journal Requirements:**

1) Please provide an Author Summary. This should appear in your manuscript between the Abstract (if applicable) and the Introduction, and should be 150-200 words long. The aim should be to make your findings accessible to a wide audience that includes both scientists and non-scientists. Sample summaries can be found on our website under Submission Guidelines:

https://journals.plos.org/plospathogens/s/submission-guidelines#loc-parts-of-a-submission

2) Your manuscript is missing the following section heading: Methods.  Please ensure all required sections are present and in the correct order. Make sure section heading levels are clearly indicated in the manuscript text, and limit sub-sections to 3 heading levels. An outline of the required sections can be consulted in our submission guidelines here:

https://journals.plos.org/plospathogens/s/submission-guidelines#loc-parts-of-a-submission

3) We notice that your supplementary figure is uploaded with the file type 'Figure'. Please amend the file type to 'Supporting Information'. Please ensure that each Supporting Information file has a legend listed in the manuscript after the references list.

4) We note that your Data Availability Statement is currently as follows: "All relevant data are in the manuscript and its Supporting information files.". Please confirm at this time whether or not your submission contains all raw data required to replicate the results of your study. Authors must share the “minimal data set” for their submission. PLOS defines the minimal data set to consist of the data required to replicate all study findings reported in the article, as well as related metadata and methods (https://journals.plos.org/plosone/s/data-availability#loc-minimal-data-set-definition).

For example, authors should submit the following data:

1) The values behind the means, standard deviations and other measures reported;

2) The values used to build graphs;

3) The points extracted from images for analysis..

Authors do not need to submit their entire data set if only a portion of the data was used in the reported study.

If your submission does not contain these data, please either upload them as Supporting Information files or deposit them to a stable, public repository and provide us with the relevant URLs, DOIs, or accession numbers. For a list of recommended repositories, please see https://journals.plos.org/plosone/s/recommended-repositories.

If there are ethical or legal restrictions on sharing a de-identified data set, please explain them in detail (e.g., data contain potentially sensitive information, data are owned by a third-party organization, etc.) and who has imposed them (e.g., an ethics committee). Please also provide contact information for a data access committee, ethics committee, or other institutional body to which data requests may be sent. If data are owned by a third party, please indicate how others may request data access.

5) Please amend your detailed Financial Disclosure statement. This is published with the article. It must therefore be completed in full sentences and contain the exact wording you wish to be published.

1) State the initials, alongside each funding source, of each author to receive each grant. For example: "This work was supported by the National Institutes of Health (####### to AM; ###### to CJ) and the National Science Foundation (###### to AM)."

2) State what role the funders took in the study. If the funders had no role in your study, please state: "The funders had no role in study design, data collection and analysis, decision to publish, or preparation of the manuscript.".

Note: If the reviewer comments include a recommendation to cite specific previously published works, please review and evaluate these publications to determine whether they are relevant and should be cited. There is no requirement to cite these works unless the editor has indicated otherwise.

**Reviewers' Comments:**

Reviewer's Responses to Questions

**Part I - Summary**

Please use this section to discuss strengths/weaknesses of study, novelty/significance, general execution and scholarship.

Reviewer #1: The manuscript by Molitor and Bogdan investigates the cell morphology of lamellocytes, an interesting class of derived immune cells induced during the Drosophila anti-parasitoid response. The reporter research analyzed the roles of the cytoskeleton and its regulators in determining lamellocyte morphology and function. Molitor and Bogdan arrived at the interesting finding that lamellocyte morphology determination is unique when compared to other classes of Drosophila immune cells, and make important strides towards identifying and characterizing this mechanism. This paper is significance in the study of host-parasite interactions and immune cell biology: their work provides a close characterization of an interesting immune cell type, which to date has largely been studied in the contexts of cell type specification and immune function. Their characterization of lamellocyte cell morphology study adds an interesting new dimension to the field.

Reviewer #2: This is an interesting study that investigates the cytoskeleton and its regulators in specialized Drosophila immune cells known as lamellocytes. The authors conduct an RNAi screen which results in the implication of a number of molecules in normal morphology of these cells. Functional validation is carried out for rac2 and fril through investigation of parasitoid wasp egg encapsulation by mutants. A weakness of the study is that the significance of negative results for the WAVE Arp2/3 are overstated. These experiments are conducted with RNAi, and the authors do not validate that the targeted proteins are indeed depleted by the RNAi methodology. To substantiate these claims, mutant alleles would need to be investigated, perhaps with MARCM clones if viability of larvae is an issue) or validation of depletion is necessary. If suitably revised the study would be of interest to the readers of PLOS Pathogens.

Reviewer #3: Cdc42 and Rac2 acts through the formin-like Frl/FMNL to control lamellocyte shape and encapsulation of parasitoid wasp eggs in Drosophila

In this manuscript the authors aim to understand which proteins functionally associated with the microfilament cytoskeleton are important in controlling the shape of lamellocytes and whether these proteins are needed for lamellocytes to wrap around the wasp egg. After describing the actin filament (and tubulin) networks in lamellocytes and their shapes and protrusions from wasp-infested hosts, the authors used RNAi lines for candidate genes and cell-specific markers to identify a putative protein complex of Frl/FMNL, Rac2, and Cdc42. They show that Frl/FMNL is localized at cortex and its localization depends on Cdc42 and Rac2 functions. All three proteins are needed for lamellocyte shape and knockdown of Frl/FMNL and Rac2 caused lamellocyte wasp encapsulation defects. These conclusions are largely supported by the data they provide. The writing, figures, tables and movies are clear, showing varied hemocyte morphologies. Quantification of the results are not explained enough to know how reproducible the results are. The novel aspects of the study could be highlighted in the context of a hypothesis.

**********

**Part II – Major Issues: Key Experiments Required for Acceptance**

Please use this section to detail the key new experiments or modifications of existing experiments that should be absolutely required to validate study conclusions.

Generally, there should be no more than 3 such required experiments or major modifications for a "Major Revision" recommendation. If more than 3 experiments are necessary to validate the study conclusions, then you are encouraged to recommend "Reject".

Reviewer #1: My only major issue is with the notable lack of experimental detail in the Methods section and figure legends. The figure legends present an N value and statistical significance for many quantitative experiments, but:

1. N is never defined. Is N the number of cells imaged, larvae dissected, or biological replicates? If N is the number of cells, how many cells were imaged per larvae per replicate? It’s essential for the authors to clarify how many independent samples were analyzed.

2. Likewise, p values are provided but the statistical tests used to determine these values are never specified.

Additionally, data are presented for measures of cell circularity and the cortex:cell body ratio of Frl staining, but the underlying methodology is not described anywhere in the manuscript. Again, the authors must provide essential details that would allow for experimental replication.

All of these issues with methods reporting make it difficult to judge the strengths and limitations of the presented data, and to assess the degree that data support the conclusions of the manuscript.

Reviewer #2: As already noted claims that WAVE-Arp 2/3 complex are not required are weakly supported by the evidence. Negative results with RNAi must be interpreted with caution. Further evidence is necessary to support these claims. Alternatively, the authors should discuss the caveats of the methodology and state that a role for this complex cannot be ruled out using RNAi.

Reviewer #3: In the following review, feedback is provided for the reviewers to consider:

The authors screen for the functional requirement of multiple proteins, predicted or known to be expressed at high levels in lamellocyte shape and function. This is reasonable starting point. To make this story more broadly interesting, the authors could articulate or define precise question(s) and consider formulating a hypothesis. For example, based on the interpretation of the data (Discussion lines 306-307), the picture that emerges is that lamellocytes are stiff, they do not appear to form lamellopodia, and their movement in vivo may be due to passive flow. Are these results novel or were they expected/unexpected? And how good is the evidence in support of this interpretation?

(1) There is significant variation in lamellocyte morphologies. Is the expression pattern of Tep4 GAL4 uniform in all lamellocyte types? If this has been studied, please mention it with reference.

(2) Does the binding of lamellocytes to Concanavalin A bias the cell population being observed?

(3) Can we say with some confidence that RNAi-mediated knockdown of these important proteins does not alter (a) hemocyte identities, and (b) expression levels of GAL4 driver(s)? Please comment on this and provide evidence.

(4) Line 134-136: … isolated lamellocytes have a very rigid disc-shaped morphology with no prominent protrusion dynamics when plated on glass surfaces. What does “very rigid” mean? If rigidity was not measured, then the authors should rephrase their interpretation to qualify what they mean and how it can be tested.

(5) Explain more clearly is the significance of the results in Fig 1 (for example tubulin results) to the rest of the paper. Do the identities of the cells change upon the introduction of these drugs/toxins?

(6) Movie S3: What are the bright signals internal to the cells? Are they ruffles or some intracellular organelles? Is it possible for the authors to interpret the internal morphology a little more?

(7) What is Cpa’s role in encapsulation?

Fig 3: Make a schematic showing cortex and body and the cortex-body ratio for a couple of different cell shapes, shown in the Figure panel, to illustrate the Result. In some cases, lamellocyte shapes are so atypical that this point needs to be clarified a bit more.

-In Fig 3A, B: the confocal microscopy image of a lamellocyte expressing the Rac/Cdc42-sensor MBT-GFP shows somewhat higher signal around the nucleus than closer to the membrane, almost complementary to the anti-Frl straining. Is this true, and if so, is it observed consistently or just in the examples provided here?

Fig 4: Methods for the protein models and interaction predictions: Clarify what is newly done in this study. Add information that tells us how reliable these interaction predictions. Is it possible to predict if and how the virulence factor in L. boulardi venom might affect these interactions?

Statistics: Add a section in the Methods describing how cells were selected (randomly or not). What was the minimum number of cells used in the analyses, which tests were done and how? How many technical and biological replicates were done for each quantification? For each result where quantification is provided, add relevant details in the Fig legend for the reader to assess the strength of the result. Biological replicates must be included for quantification and subsequent publication.

**********

**Part III – Minor Issues: Editorial and Data Presentation Modifications**

Please use this section for editorial suggestions as well as relatively minor modifications of existing data that would enhance clarity.

Reviewer #1: For the lamellocyte: wasp egg interaction studies (in lines 143-147), how were the wasp eggs collected? It’s been previously established (e.g., Russo et al., Parasitology, 1996; Mortimer et al., Plos Pathogens, 2012) that plasmatocytes bind directly to the wasp egg surface, and that the lamellocytes are more likely to interact with the plasmatocyte layer in capsule formation. Depending on the egg collection, this plasmatocyte layer may not be present, e.g. if eggs are isolated directly from the ovaries. This method should be more clearly specified, and the conclusions considered carefully if the cell layer is absent. Also, lines 309-310 refer to lamellocyte-egg contacts and may need to be amended.

Additionally, the descriptions of Suppl. movies M4+M5 are confusing (lines 783-790). It’s unclear how the conclusions are reached. These interactions seem very short lived, in contrast to capsule formation. In M4 the parasitoid egg also seems to be considerably bigger than the purported wasp larva in M5 even though the images taken at the same scale. M5 also appears to show a wasp egg with the same size and appearance of the egg in M4.

It would be helpful to provide more context for some of the reported screen results. For instance, lines 211-212 list several genes with phenotypes. Are these genes known interactors of Frl? Is this a new connection between these genes?

Similarly, lines 213-214 describe two Diaphanous-related formin genes with no morphological phenotypes. The text previously described Frl as the sole member of the Diaphanous-related formin subfamily in Drosophila (lines 207-208). This relationship needs to be clarified.

In line 354, I think that saying that use of structure simulations “confirmed” binding interactions is misleading. Overall, I’m not convinced that the structure simulations add anything to the manuscript, and could be removed, but at a minimum they should be better described.

I also noted some issues with terminology.

First, the term avirulent is used, e.g., line 269. It is not accurate to say that this wasp strain is avirulent, given that the data show that most of the wasps are evading encapsulation and hatching in the host (lines 270-271). The use of avirulent would imply that the wasps have no immune evasive (or other) activity against the host.

Similarly, I don’t think it’s correct to characterize the eggs as properly encapsulated in line 274. The described interactions would not be sufficient to conclude that encapsulation is occurring, again particularly given that most of the wasps are not being encapsulated. Proper encapsulation would specifically refer to a wasp egg completely surrounded by a melanized capsule and killed by this immune reaction. I completely agree with the authors that the differences in lamellocyte interaction with the wasp egg between genotypes are interesting and likely functionally relevant, but this alternative use of established terminology is misleading.

Comments on the figures and legends:

As noted above, replication and statistical testing information need to be added to all figures where appropriate.

Figure 1: Aspect is misspelled in the figure.

The use of “cell size” (lines 677,679) is potentially confusing. It would be better to stick with cell area as the data are presented.

Figure 2: A: What is meant by “% expressed”? It could be a normalized expression value, or the number of expressing cells per cluster, so please make the description more precise.

Also, please consider recoloring the average expression value, a single-color gradient is very difficult for many people with vision issues to interpret. Personally, I see the scale as grey for -1.0 and as a single red color for all values between -0.5 to 1.0.

Figure 4: The reader’s ability to interpret panels B and C would be greatly aided by displaying all of channels individually as shown for A.

As noted above, I’m not sure that the structure models presented in F-G’ add to the story. At a minimum, the use of color in the model should be explained in the legend.

Suppl. Figure 1: A: the grey boxed genes are not explained by the legend. I’m assuming these genes were tested but no phenotype was seen, but it’s not clear. It would also help assessment of the screen if number of replicates or individuals screened, and/or the criteria for phenotype determination were reported.

Suppl. Movies: Several of the movies have different display titles within a viewer (I used QuickTime Player for Mac). It’s not a big deal, but for instance movie M6 is titled movie M3 in the viewer, so I wanted to pass this observation along in case it becomes confusing.

Reviewer #2: (No Response)

Reviewer #3: Scale bars: Some panels are lacking scale bars, or maybe they are just not visible?

Typos:

-Line 135 has a typo (glass, not glas).

-Lines 138 and 152: The spelling of Concanavalin A is incorrect

-Line 150 and other places: The genotype Hoptum-l should be hopTum-l (of course with italics and superscript, as already used)

-Line 722: “ist” stained should be “is” stained

-Please check in all other places

While the use of gene symbol abbreviations ((Frl/FMNL) is fine, it feels a little tedious. The authors could consider picking one or the other, after having defined them in the Introduction. Consider rephrasing the Title to see if it works in the absence of the gene symbols. It might enhance the impact of the study.

**********

PLOS authors have the option to publish the peer review history of their article (what does this mean?). If published, this will include your full peer review and any attached files.

If you choose “no”, your identity will remain anonymous but your review may still be made public.

**Do you want your identity to be public for this peer review?**  For information about this choice, including consent withdrawal, please see our Privacy Policy.

Reviewer #1: No

Reviewer #2: No

Reviewer #3: No

[NOTE: If reviewer comments were submitted as an attachment file, they will be attached to this email and accessible via the submission site. Please log into your account, locate the manuscript record, and check for the action link "View Attachments". If this link does not appear, there are no attachment files.]

**Figure resubmission:**

-->While revising your submission, we strongly recommend that you use PLOS’s NAAS tool (https://ngplosjournals.pagemajik.ai/artanalysis) to test your figure files. NAAS can convert your figure files to the TIFF file type and meet basic requirements (such as print size, resolution), or provide you with a report on issues that do not meet our requirements and that NAAS cannot fix.

After uploading your figures to PLOS’s NAAS tool - https://ngplosjournals.pagemajik.ai/artanalysis, NAAS will process the files provided and display the results in the "Uploaded Files" section of the page as the processing is complete. If the uploaded figures meet our requirements (or NAAS is able to fix the files to meet our requirements), the figure will be marked as "fixed" above. If NAAS is unable to fix the files, a red "failed" label will appear above. When NAAS has confirmed that the figure files meet our requirements, please download the file via the download option, and include these NAAS processed figure files when submitting your revised manuscript.

**Reproducibility:**

To enhance the reproducibility of your results, we recommend that authors of applicable studies deposit laboratory protocols in protocols.io, where a protocol can be assigned its own identifier (DOI) such that it can be cited independently in the future. Additionally, PLOS ONE offers an option to publish peer-reviewed clinical study protocols. Read more information on sharing protocols at https://plos.org/protocols?utm_medium=editorial-email&utm_source=authorletters&utm_campaign=protocols

# Author comment: Author response to Decision Letter 1

**DOI:** 10.1371/journal.ppat.1014440.r002

13 May 2026

Attachment Submitted filename: Response to reviewers 11.05.26..docx

# Peer review: Decision Letter 1

**DOI:** 10.1371/journal.ppat.1014440.r003

**Authors:** Jeffrey Dvorin, Robert Unckless

26 Jun 2026

PPATHOGENS-D-25-03187R1

Cdc42 and Rac2 act through the formin-like Frl/FMNL to control lamellocyte shape and encapsulation of parasitoid wasp eggs in Drosophila

PLOS Pathogens

Dear Dr. Bogdan,

Thank you for submitting your manuscript to PLOS Pathogens. After careful consideration, we feel that it has merit but does not fully meet PLOS Pathogens's publication criteria as it currently stands. Therefore, we invite you to submit a revised version of the manuscript that addresses the points raised during the review process.

Please submit your revised manuscript by Aug 25 2026 11:59PM. If you will need more time than this to complete your revisions, please reply to this message or contact the journal office at plospathogens@plos.org. When you're ready to submit your revision, log on to https://www.editorialmanager.com/ppathogens/ and select the 'Submissions Needing Revision' folder to locate your manuscript file.

Please include the following items when submitting your revised manuscript:

* A letter that responds to each point raised by the editor and reviewer(s). You should upload this letter as a separate file labeled 'Response to Reviewers'. This file does not need to include responses to any formatting updates and technical items listed in the 'Journal Requirements' section below.

* A marked-up copy of your manuscript that highlights changes made to the original version. You should upload this as a separate file labeled 'Revised Manuscript with Track Changes'.

* An unmarked version of your revised paper without tracked changes. You should upload this as a separate file labeled 'Manuscript'.

If you would like to make changes to your financial disclosure, competing interests statement, or data availability statement, please make these updates within the submission form at the time of resubmission. Guidelines for resubmitting your figure files are available below the reviewer comments at the end of this letter.

As the corresponding author, your ORCID iD is verified in the submission system and will appear in the published article. PLOS supports the use of ORCID, and we encourage all coauthors to register for an ORCID iD and use it as well. Please encourage your coauthors to verify their ORCID iD within the submission system before final acceptance, as unverified ORCID iDs will not appear in the published article. Only the individual author can complete the verification step; PLOS staff cannot verify ORCID iDs on behalf of authors.

We look forward to receiving your revised manuscript.

Kind regards,

Robert L. Unckless, Ph.D.

Academic Editor

PLOS Pathogens

Jeffrey Dvorin

Section Editor

PLOS Pathogens

Sumita Bhaduri-McIntosh

Editor-in-Chief

PLOS Pathogens

orcid.org/0000-0003-2946-9497

Michael Malim

Editor-in-Chief

PLOS Pathogens

orcid.org/0000-0002-7699-2064

**Additional Editor Comments (if provided):**

Two expert reviewers have considered your revised manuscript. Both felt that there are still some clarifications that need to be made prior to publication. These revisions should focus on total transparency in terms of limitations in the methods (R2) and explanation of sample size, etc. (R1). We look forward to your revised manuscript. We are providing a decision of "Minor Revision" but note that these are crucial items that need to be addressed.

**Journal Requirements:**

If the reviewer comments include a recommendation to cite specific previously published works, please review and evaluate these publications to determine whether they are relevant and should be cited. There is no requirement to cite these works unless the editor has indicated otherwise.

1) Please ensure that the CRediT author contributions listed for every co-author are completed accurately and in full.

At this stage, the following Authors/Authors require contributions: Hermann Schillers. Please ensure that the full contributions of each author are acknowledged in the "Add/Edit/Remove Authors" section of our submission form.

The list of CRediT author contributions may be found here: https://journals.plos.org/plospathogens/s/authorship#loc-author-contributions

2)We have amended your Competing Interest statement to comply with journal style. We kindly ask that you double check the statement and let us know if anything is incorrect.

**Reviewers' Comments:**

Reviewer's Responses to Questions

**Part I - Summary**

Please use this section to discuss strengths/weaknesses of study, novelty/significance, general execution and scholarship.

Reviewer #1: This manuscript by Molitor et al., provides an interesting and well-supported characterization of the Drosophila lamellocyte, an interesting yet understudied immune cell type. The authors have done a commendable job of addressing the reviewers' concerns, and the additional experiments and clarifications have significantly improved their manuscript. I have one concern about the GST-binding experiments, a few minor comments for clarification.

Reviewer #3: The authors have made considerable efforts to address reviewer comments and the revised manuscript is significantly stronger. Additional points are below for the authors to consider to strengthen the manuscript further.

**********

**Part II – Major Issues: Key Experiments Required for Acceptance**

Please use this section to detail the key new experiments or modifications of existing experiments that should be absolutely required to validate study conclusions.

Generally, there should be no more than 3 such required experiments or major modifications for a "Major Revision" recommendation. If more than 3 experiments are necessary to validate the study conclusions, then you are encouraged to recommend "Reject".

Reviewer #1: Lines 323-324. According to the Methods (lines 553-555), the GST constructs for Cdc42 and Rac2 are not equivalent (i.e., that the activating Cdc42[V12] mutation was used, alongside the wild type Rac2). This difference does not seem to be mentioned in the Results or Discussion, despite this change potentially influencing binding, particularly given the result that Cdc42[v12] binds to Frl/FMNL whereas Rac2[WT] doesn't.

Reviewer #3: 1. Quantification and replication: Maybe I missed this but I did not find how many biological replicates were performed for each experiment. Is a cell considered a technical replicate of another cell? Is a larva from the same vial a technical or biological replicate? The authors should clarify this point more rigorously as this information was requested by all reviewers. The manuscript should not be published without biological replicates for all experiments, each including multiple technical replicates. Statistical analysis should then be performed from data derived from these replicates.

Example of the kind of information that is missing: This is text from Fig. 1A legend: Elasticity of cells given as the Young`s modulus. Indentation experiments revealed that partially spread lamellocytes are stiffer than fully spread lamellocytes. N = 40 (plasmatocytes), 74 (partially spread lamellocytes) and 180 (fully spread lamellocytes) force-distance curves from three independent preparations; One-way ANOVA with Kruskal-Wallis test, ***: p < 0.001.

2. Is the number of cells presented derived from all three preparations? Were preparations made from one animal or multiple animals in the same vial or multiple animals from different vials? The authors need to be clear about how they define a replicate. Explain in one sentence how Young’s modulus informs us of cell elasticity. The values and variation in the Young’s modulus numbers need to be mentioned in the Results sections.

Similar feedback needs to be applied to all experiments, whether they were quantified or not.

3. Starting from the Abstract (Remarkably, cell spreading does not depend on WAVE-Arp2/3-branched actin filament nucleation but rather requires the formin Frl (also known as FMNL) downstream of Rac2 and Cdc42 signaling.), the authors imply that these two mechanisms are alternative to each other for the same function (cell spreading in this case). But in the Discussion, they discuss this issue in the context of cell protrusion and cell migration. From a cell biology point-of-view aren’t these behaviors different and assay dependent? It would be helpful for the readers if the authors qualify these terms in the context of the assays and the biology/physiology of the cell.

4. Instead of saying “…. WAVE-Arp2/3 function has no important role in lamellocyte morphology, I would suggest rephrasing to say “ Arp2/3 has no apparent or detectable role/function in…”.

5. The movies show that the number of lamellocytes around the wasp egg is very low; at most one lamellocyte is shown in representative movies. The authors need to acknowledge that these few lamellocytes (how many cells observed in movies from how many experiments?) may have partially escaped the effects of the venom.

6. For the pulldown experiments, describe the methods for protein expression and include references (or sources) for constructs (e.g., pUAST attB GFP-Frl). Separate this information from the steps used in the pull-down experiment itself.

For the Western blot Methods section, add more details to the antibodies used. Mention conditions for staining the Western blots.

In the Western blot Figure panel, show more clearly which antigens were tagged with GFP so it is easy to interpret the result. Why are the two Western blots in separate pieces? Were the markers not run in the same gel as the three samples in the right-most lanes?

7. Line 501-502: For fixation, eggs were collected in a drop of medium on a silane prep slide. What medium? Give details of the “silane prep slide”

8. The genotype Hoptum-l: According to Flybase, the symbol for the hopscotch gene is hop (h not capitalized). The Tum-l allele is dominant, hence the t in tum-l should be capitalized. Not the other way round.

9. Movies 9 and 10 are dispensable.

10. In general, the quality of the Discussion can be improved. Right now, various results are mentioned in isolation of each other, but not synthesized in a cohesive picture. For example, the issue of lamellocyte rigidity is not tied to the molecular data in the manuscript. Is it known for other cell types how actin modulators affect cell rigidity? The authors should try to include as many results in the discussion as possible. The discussion of lamellolysin in L. heterotoma is not relevant to this manuscript as it is not expected to be present in L. boulardi. The authors should consider removing it.

**********

**Part III – Minor Issues: Editorial and Data Presentation Modifications**

Please use this section for editorial suggestions as well as relatively minor modifications of existing data that would enhance clarity.

Reviewer #1: The protocol for measuring circularity is still not clearly defined, at least not for my understanding. I'm assuming it is linked to the cortical measurements, but the word "circularity" doesn’t appear in the methods section so I'm still not able to follow the approach. I think this just needs a more explicit description.

Line 338 includes the idea that “heterodimers might act as a bridge, forming Frl/FMNL complexes with specialized cellular roles.” The predicted ability to bind to heterodimers is intriguing, but I'm not sure what the authors' statement means by "act[ing] as a bridge". This just needs a text clarification.

Reviewer #3: Please see above.

**********

PLOS authors have the option to publish the peer review history of their article (what does this mean?). If published, this will include your full peer review and any attached files.

If you choose “no”, your identity will remain anonymous but your review may still be made public.

**Do you want your identity to be public for this peer review?**  For information about this choice, including consent withdrawal, please see our Privacy Policy.

Reviewer #1: No

Reviewer #3: No

[NOTE: If reviewer comments were submitted as an attachment file, they will be attached to this email and accessible via the submission site. Please log into your account, locate the manuscript record, and check for the action link "View Attachments". If this link does not appear, there are no attachment files.]

**Figure resubmission:**

-->While revising your submission, we strongly recommend that you use PLOS’s NAAS tool (https://ngplosjournals.pagemajik.ai/artanalysis) to test your figure files. NAAS can convert your figure files to the TIFF file type and meet basic requirements (such as print size, resolution), or provide you with a report on issues that do not meet our requirements and that NAAS cannot fix.

After uploading your figures to PLOS’s NAAS tool - https://ngplosjournals.pagemajik.ai/artanalysis, NAAS will process the files provided and display the results in the "Uploaded Files" section of the page as the processing is complete. If the uploaded figures meet our requirements (or NAAS is able to fix the files to meet our requirements), the figure will be marked as "fixed" above. If NAAS is unable to fix the files, a red "failed" label will appear above. When NAAS has confirmed that the figure files meet our requirements, please download the file via the download option, and include these NAAS processed figure files when submitting your revised manuscript.

**Reproducibility:**

To enhance the reproducibility of your results, we recommend that authors of applicable studies deposit laboratory protocols in protocols.io, where a protocol can be assigned its own identifier (DOI) such that it can be cited independently in the future. Additionally, PLOS ONE offers an option to publish peer-reviewed clinical study protocols. Read more information on sharing protocols at https://plos.org/protocols?utm_medium=editorial-email&utm_source=authorletters&utm_campaign=protocols

# Author comment: Author response to Decision Letter 2

**DOI:** 10.1371/journal.ppat.1014440.r004

1 Jul 2026

Attachment Submitted filename: Response to Reviewers 30.06.26.docx

# Editor report: Decision Letter 2

**DOI:** 10.1371/journal.ppat.1014440.r005

**Authors:** Jeffrey Dvorin, Robert Unckless

3 Jul 2026

Dear Dr. Bogdan,

We are pleased to inform you that your manuscript 'Cdc42 and Rac2 act through the formin-like Frl/FMNL to control lamellocyte shape and encapsulation of parasitoid wasp eggs in Drosophila' has been provisionally accepted for publication in PLOS Pathogens.

Before your manuscript can be formally accepted you will need to complete some formatting changes, which you will receive in a follow up email. A member of our team will be in touch with a set of requests.

Please note that your manuscript will not be scheduled for publication until you have made the required changes, so a swift response is appreciated.

IMPORTANT: The editorial review process is now complete. PLOS will only permit corrections to spelling, formatting or significant scientific errors from this point onwards. Requests for major changes, or any which affect the scientific understanding of your work, will cause delays to the publication date of your manuscript.

Should you, your institution's press office or the journal office choose to press release your paper, you will automatically be opted out of early publication. We ask that you notify us now if you or your institution is planning to press release the article. All press must be co-ordinated with PLOS.

Thank you again for supporting Open Access publishing; we are looking forward to publishing your work in PLOS Pathogens.

Best regards,

Robert L. Unckless, Ph.D.

Academic Editor

PLOS Pathogens

Jeffrey Dvorin

Section Editor

PLOS Pathogens

Sumita Bhaduri-McIntosh

Editor-in-Chief

PLOS Pathogens

orcid.org/0000-0003-2946-9497

Michael Malim

Editor-in-Chief

PLOS Pathogens

orcid.org/0000-0002-7699-2064

***********************************************************

Thank you for carefully addressing reviewer comments. I look forward to seeing this in print.

Reviewer Comments (if any, and for reference):

# Editor report: Acceptance letter

**DOI:** 10.1371/journal.ppat.1014440.r006

**Authors:** Jeffrey Dvorin, Robert Unckless

Dear Dr. Bogdan,

We are delighted to inform you that your manuscript, "Cdc42 and Rac2 act through the formin-like Frl/FMNL to control lamellocyte shape and encapsulation of parasitoid wasp eggs in Drosophila," has been formally accepted for publication in PLOS Pathogens.

We have now passed your article onto the PLOS Production Department who will complete the rest of the pre-publication process. All authors will receive a confirmation email upon publication.

The corresponding author will soon be receiving a typeset proof for review, to ensure errors have not been introduced during production. Please review the PDF proof of your manuscript carefully, as this is the last chance to correct any scientific or type-setting errors. Please note that major changes, or those which affect the scientific understanding of the work, will likely cause delays to the publication date of your manuscript. Note: Proofs for Front Matter articles (Pearls, Reviews, Opinions, etc...) are generated on a different schedule and may not be made available as quickly.

Soon after your final files are uploaded, the early version of your manuscript, if you opted to have an early version of your article, will be published online. The date of the early version will be your article's publication date. The final article will be published to the same URL, and all versions of the paper will be accessible to readers.

For Research Articles, you will receive an invoice from PLOS for your publication fee after your manuscript has reached the completed accept phase. If you receive an email requesting payment before acceptance or for any other service, this may be a phishing scheme. Learn how to identify phishing emails and protect your accounts at https://explore.plos.org/phishing.

Thank you again for supporting open-access publishing; we are looking forward to publishing your work in PLOS Pathogens.

Best regards,

Sumita Bhaduri-McIntosh

Editor-in-Chief

PLOS Pathogens

orcid.org/0000-0003-2946-9497

Michael Malim

Editor-in-Chief

PLOS Pathogens

orcid.org/0000-0002-7699-2064
